# Iodine(I) and Silver(I) Complexes Incorporating 3-Substituted Pyridines

**DOI:** 10.1021/acsomega.3c03097

**Published:** 2023-06-21

**Authors:** Kari Rissanen, Jas S. Ward

**Affiliations:** Department of Chemistry, University of Jyvaskyla, Jyväskylä 40014, Finland

## Abstract

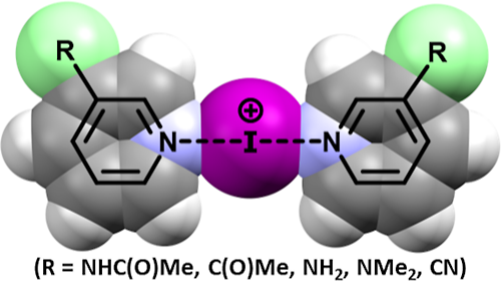

Building upon the first report of a 3-acetaminopyridine-based iodine(I) complex (**1b**) and its unexpected reactivity toward ^t^BuOMe, several new 3-substituted iodine(I) complexes (**2b–5b**) have been synthesized. The iodine(I) complexes were synthesized from their analogous silver(I) complexes (**2a–5a**) via a silver(I) to iodine(I) cation exchange reaction, incorporating functionally related substituents as 3-acetaminopyridine in **1b**; 3-acetylpyridine (3-Acpy; **2**), 3-aminopyridine (3-NH_2_py; **3**), and 3-dimethylaminopyridine (3-NMe_2_py; **4**), as well as the strongly electron-withdrawing 3-cyanopyridine (3-CNpy; **5**), to probe the possible limitations of iodine(I) complex formation. The individual properties of these rare examples of iodine(I) complexes incorporating 3-substituted pyridines are also compared to each other and contrasted to their 4-substituted counterparts which are more prevalent in the literature. While the reactivity of **1b** toward etheric solvents could not be reproduced in any of the functionally related analogues synthesized herein, the reactivity of **1b** was further expanded to a second etheric solvent. Reaction of *bis*(3-acetaminopyridine)iodine(I) (**1b**) and ^i^Pr_2_O gave [3-acetamido-1-(3-iodo-2-methylpentan-2-yl)pyridin-1-ium]PF_6_ (**1d**), which demonstrated potentially useful C–C and C–I bond formation under ambient conditions.

## Introduction

Halogen bonding enjoys being one of the most studied types of intermolecular interactions after hydrogen bonding, and as such has been deftly employed to construct a myriad of magnetic, porous, phosphorescent, and liquid-crystalline materials toward applications such as biomolecular engineering, chemical separations, and ion-pair recognition.^1−3^ The incorporation of halogen bond donors into polymers has led to the development of topochemical polymerization, molecularly-imprinted polymers, functional-/stimuli-responsive polymeric materials,^1,2,4^ and, most recently, shape-memory polymers.^5^ A main advantage of halogen bonding is its highly directional nature, owing to its electronic origin as a σ–hole interaction (*i.e.*, the tightly confined electropositive region along the axis of the halogen bond donor’s R–X bond),^6^ which has found great utility in the construction of a variety of supramolecular architectures.^7−14^

The epitome of halogen bonding is the linear halogen(I) (also termed halonium) complexes,^15,16^ which comprise a halenium ion (X^+^; X = Cl, Br, and I) and a pair of stabilizing Lewis bases (L; commonly nitrogen-based aromatic ligands such as pyridine), [L–X–L]^+^. The stability of halogen(I) complexes follows the trend: I > Br ≫ Cl,^17,18^ which is reflected in the number of solid-state examples reported for each type,^19^ with Barluenga’s reagent, *bis*(pyridine)iodine(I) tetrafluoroborate, being the paradigm of iodine(I) complexes due to its widespread use in a multitude of organic transformations as a mild iodinating and oxidizing reagent.^20−24^ Halogen(I) complexes, [L–X–L]^+^, feature a 3-center 4-electron (**3c–4e**) bond, the symmetric nature of which has been confirmed computationally and in solution.^25,26^ The negatively charged [O–I–O]^−^ complexes are also known to have applications as organic reagents,^27^ and recently the ability to instigate asymmetry in the halogen bonding, via hydrogen bonding with one of the two saccharinato ligands, in the analogous [N–I–N]^−^ complexes has been demonstrated.^28^ Interest in halogen(I) chemistry has been steadily increasing in recent years, with a whole slew of recent advances being reported, including the first examples of unrestrained heteroleptic,^29−31^ hierarchical,^32^ and nucleophilic interactions of iodine(I) complexes,^33−35^ as well as the resurgence of (isolable) non-chiral^24,36−38^ and chiral^39^ carbonyl hypoiodites in the context of being halogen-bonded iodine(I) complexes.

**Scheme 1:**
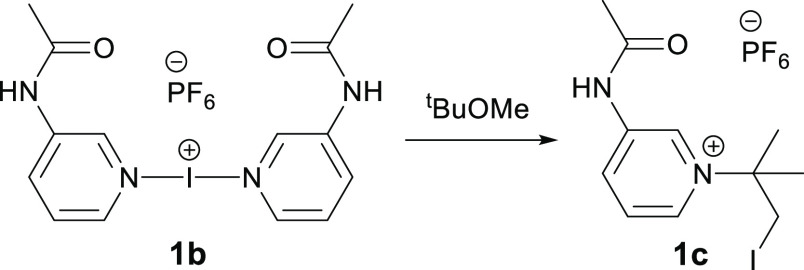
Reactivity of the First Hierarchical Iodine(I) Complex with ^t^BuOMe

The aforementioned first report of a hierarchical iodine(I) complex, [I(3-AcNHpy)_2_]PF_6_ (**1b**; 3-AcNHpy = 3-acetaminopyridine),^32^ which was concomitantly the first report of an iodine(I) species incorporating a 3-substituted pyridine as the stabilizing Lewis base, displayed unexpected reactivity toward the ^t^BuOMe to give the product **1c** (Scheme 1). However, the origin and scope of this reactivity was not fully explored and will be further investigated herein.

## Results and Discussion

**Scheme 2:**
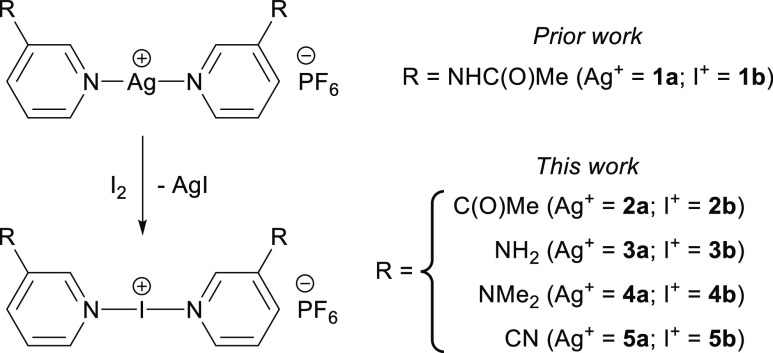
Synthesis of Iodine(I) Complexes (**2b–5b**) from Their Respective Silver(I) Complexes (**2a–5a**) via Cation Exchange, Including the Only Prior Literature Example of an Iodine(I) Complex Incorporating a 3-Substituted Pyridine (**1b**) and Its Respective Silver(I) Precursor (**1a**)^32^

A range of 3-substituted iodine(I) complexes of the form [L–I–L]PF_6_ (L = 3-substituted pyridine; **2b–5b**) were synthesized incorporating functionally analogous substituents that would provide insights into the unexpected reactivity of **1b**, which included 3-acetylpyridine (**2**), 3-aminopyridine (**3**), and 3-dimethylaminopyridine (**4**). The strongly electron-withdrawing 3-cyanopyridine (**5**) was also included to provide a more comprehensive overview of the scope of iodine(I) complexes incorporating 3-substituted pyridines, in contrast to the weakly electron-withdrawing (**1**/**2**) or strongly electron-donating (**3**/**4**) other substituents. The synthesis of the iodine(I) complexes (**2b–5b**) was achieved via cation exchange of their respective silver(I) complexes,^40,41^ [L–Ag–L]PF_6_ (**2a–5a**), which themselves were straightforwardly prepared by the addition of two equivalents of each ligand (**2–5**) to one equivalent of AgPF_6_ (Scheme 2).

All of the silver(I) and iodine(I) complexes were studied by ^1^H and ^1^H–^15^N heteronuclear multiple bond correlation (HMBC) NMR studies in CD_3_CN and, where possible due to its prevalence in prior literature examples, in CD_2_Cl_2_, with the exception of **5b** for which satisfactory NMR studies could not be performed prior to degradation, as had been observed for its 4-substituted analogue [I(4-cyanopyridine)]PF_6_.^24^^1^H NMR spectroscopy following reaction progression, starting from the uncoordinated ligands (**2**–**5**) to the silver(I) complexes (**2a**–**5a**) and finally to the iodine(I) complexes (**2b**–**5b**), all demonstrated the previously observed general trend of becoming more deshielded.^41−43^ The largest shift of uncoordinated ligand to iodine(I) complex of 0.66 ppm was observed for the conversion of **3** to **3b**, and the smallest shift of 0.05 ppm was observed between **4** to **4b**.

The ^15^N NMR chemical shifts were determined via ^1^H–^15^N HMBC studies, as these have been shown to be particularly responsive and characteristic in halogen(I) chemistry for the pyridinic nitrogen atoms (Table 1).^29,36^ Those of the uncoordinated ligands (**2**–**5**) in CD_3_CN all fell within the extremely narrow range of −63.2 (**5**) to −65.3 (**2**) ppm. A similarly narrow and characteristic range of 2.0 ppm was observed for the iodine(I) complexes, again in CD_3_CN, of −175.9 (**2b**), −173.9 (**3b**), and −174.0 (**4b**) ppm, which compared well to that reported for **1b** of −174.5 ppm (also in CD_3_CN). For the two iodine(I) complexes that could also be studied in CD_2_Cl_2_, **2b** (−175.2 ppm) and **4b** (−173.4 ppm), the ^15^N NMR resonances showed negligible differences of 0.7 and 0.6 ppm (from the CD_3_CN-recorded chemical shifts), respectively, supporting prior studies indicating the oblivious nature of the iodine(I) center toward external interactions such as with counterions^40^ or as found here with potentially coordinating solvents like MeCN. The possibility of MeCN coordinating to iodine(I) centers has been previously explored, and discounted, by Erdélyi and co-workers due to the orbital structure of the iodine(I).^44^

**Table 1:** Comparison of ^15^N NMR Chemical Shifts in CD_3_CN or CD_2_Cl_2_ (When Possible) of the Pyridinic Nitrogen Atoms of the 3-Substituted Pyridine Ligands and Their Silver(I) and Iodine(I) Complexes (in ppm)

compound/complex	pyridinic ^15^N NMR chemical shift(s) (δN)^a^		
	ligands	silver(I) complexes (**a**)	iodine(I) complexes (**b**)
3-AcNHpy (**1**)^32^	–63.7	–85.8	–174.5
3-Acpy (**2**)	–65.3 [−66.4^b^]	–83.6 [bc]	–175.9 [−175.2^b^]
3-NH_2_py (**3**)	–64.0 [bc]	–98.8 [bc]	–173.9 [bc]
3-NMe_2_py (**4**)	–65.3 [−67.4^b^]	–103.3 [−132.3^b^]	–174.0 [−173.4^b^]
3-CNpy (**5**)	–63.2 [−63.5^b^]	–68.8 [bc]	not observed [not observed^b^]

a The accuracy of reported ^15^N NMR chemical shifts is ±0.6 or ±0.8 ppm.

b Recorded in CD_2_Cl_2_.

c Could not be observed due to poor solubility in the solvent.

The silver(I) complexes, however, displayed much greater variety in their pyridinic ^15^N NMR chemical shifts, with a much larger range of values (in CD_3_CN) from −68.8 ppm (**5a**) to −103.3 ppm (**4a**). In contrast to halogen(I), the silver(I) cations are amenable to additional interactions supplemental to their linear coordination geometry, such as from weakly coordinating anions like triflate or potentially coordinating solvents like MeCN (as was reported for **1a**).^19,32,43^ The necessity of using more strongly polar deuterated solvents like MeCN for the NMR studies, however, does complicate analysis of the solution-state NMR data for complexes **2a**–**5a**, given that the observed ^15^N NMR chemical shifts will be of an equilibrium of a complicated mixture of MeCN-solvated silver(I) complexes that exist in solution, *e.g.*, **2a**·(MeCN)_*n*_ (*n* = 0–4).^45,46^ Unfortunately, the diminished organic-solvent solubility that results from the Ag^+^ cations proclivity to facilitate more intermolecular interactions only enabled **4a** to be studied in CD_2_Cl_2_, with the ^15^N NMR resonance of −132.3 ppm being significantly different from the one taken in CD_3_CN (cf. −103.3 ppm). This difference can be explained by the CD_3_CN solvent coordinating to the silver(I), which the essentially non-coordinating CD_2_Cl_2_ solvent is incapable of, as demonstrated recently by solid-state NMR studies of similar silver(I) complexes.^46^

Put in the context of previously reported analogous series of ligand (L) to Ag^+^ to I^+^ conversions for the structurally isomeric 4-substituted pyridines, meaningful electronic structure comparisons can be drawn. The 4-substituted analogues of iodine(I) complexes **3b** (with different anions other than PF_6_) and **5b**, [I(4-aminopyridine)_2_][anion] (anion = Cl, IBr_2_, I_7_) and [I(4-cyanopyridine)_2_]PF_6_, are known;^24,47^ however, no ^15^N NMR data was reported for either of these iodine(I) complexes for comparison. Fortunately, the comparative NMR data for 4-dimethylaminopyridine (4-NMe_2_py) is available, for which the pyridinic ^15^N NMR resonances in CD_2_Cl_2_ were −108.9 (ligand), −169.8 (silver(I) complex), and −217.8 (iodine(I) complex) ppm, and when compared to those of the analogous series (also in CD_2_Cl_2_) of **4** (−67.4 ppm), **4a** (−132.3 ppm), and **4b** (−173.4 ppm), **4**–**4b** were all observed to be more deshielded (Δδ_N_) by 41.5, 37.5, and 44.4 ppm, respectively. These differences demonstrate significantly modulated electronic structures in solution, as would be expected for the resonance-favored coordination of 4-NMe_2_py and the resonance-disfavored coordination of 3-NMe_2_py.

**Figure 1:**
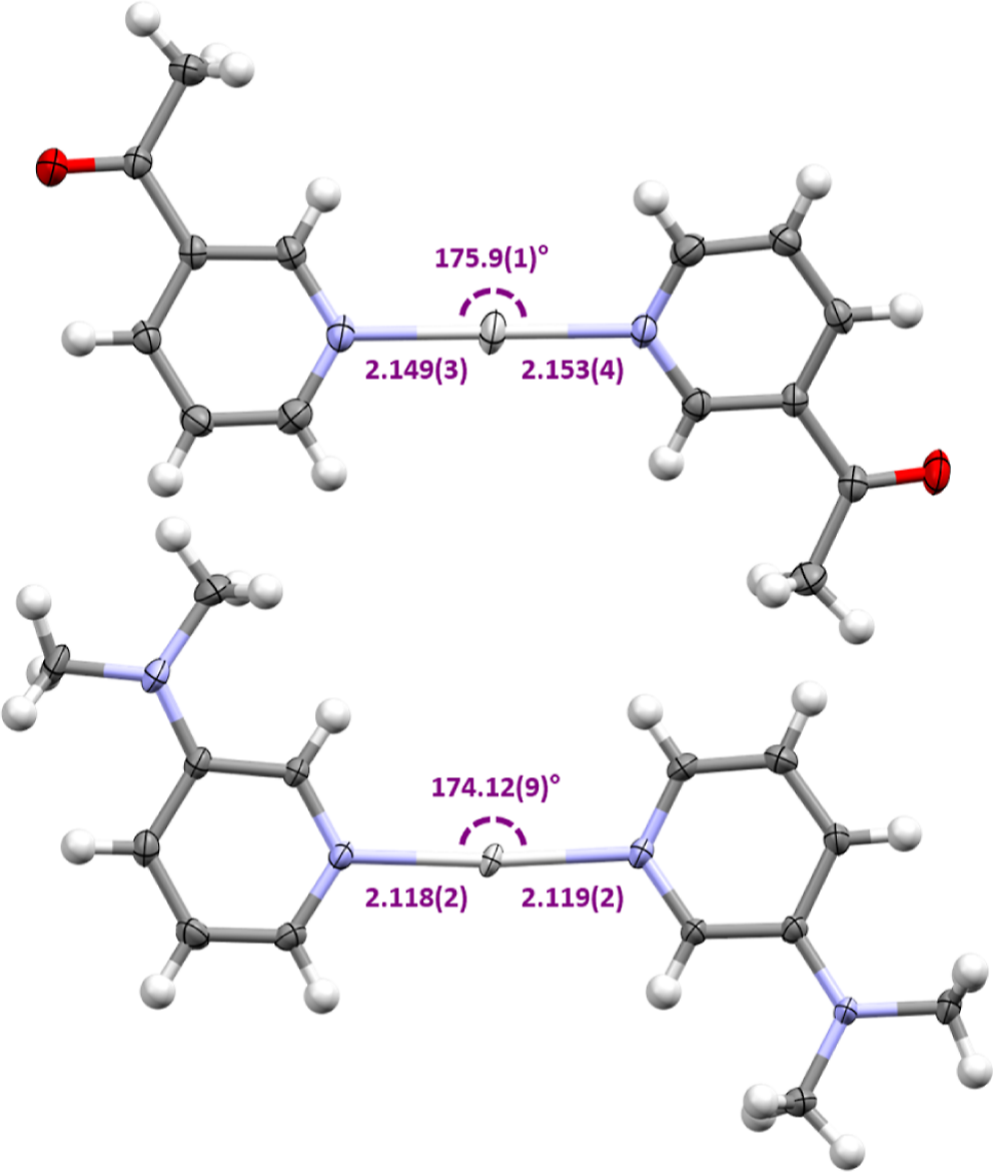
Crystal structures of the 2-coordinate silver(I) complexes **2a** (top) and **4a** (bottom) annotated with their Ag–N bond lengths (Å) and N–Ag–N bond angles (thermal ellipsoids at 50% probability; PF_6_ anions are omitted for clarity).

The solid-state structures were also determined for all silver(I) (**2a**–**5a**) and iodine(I) complexes (**2b**–**5b**) by single-crystal X-ray diffraction. Unlike their halogen(I) analogues with their strong preference for 2-coordinate linear geometries, the silver(I) complexes display a myriad of coordination geometries.^19^ Nevertheless, complexes **2a** and **4a** were observed as the linear 2-coordinate complexes with Ag–N bond lengths of 2.149(3)/2.153(4) Å and 2.118(2)/2.119(2) Å and N–Ag–N angles of 175.9(1) and 174.12(9)°, respectively (Figure 1). Complex **2a** was found to be a discrete salt, with the only intermolecular interaction being the acetyl oxygen atoms coordinating to the silver(I) center of a neighboring cation with a Ag···O distance of 2.866(3) Å. On the other hand, **4a** was found to be an argentophilic dimer (Ag···Ag = 3.2805(3) Å) that also possessed a close contact between the nitrogen atom of a neighboring NMe_2_ group and the Ag^+^ center (Ag···N = 3.217(3) Å) that was just below the sum of van der Waals radii (Ag + N = 3.27 Å).

**Figure 2:**
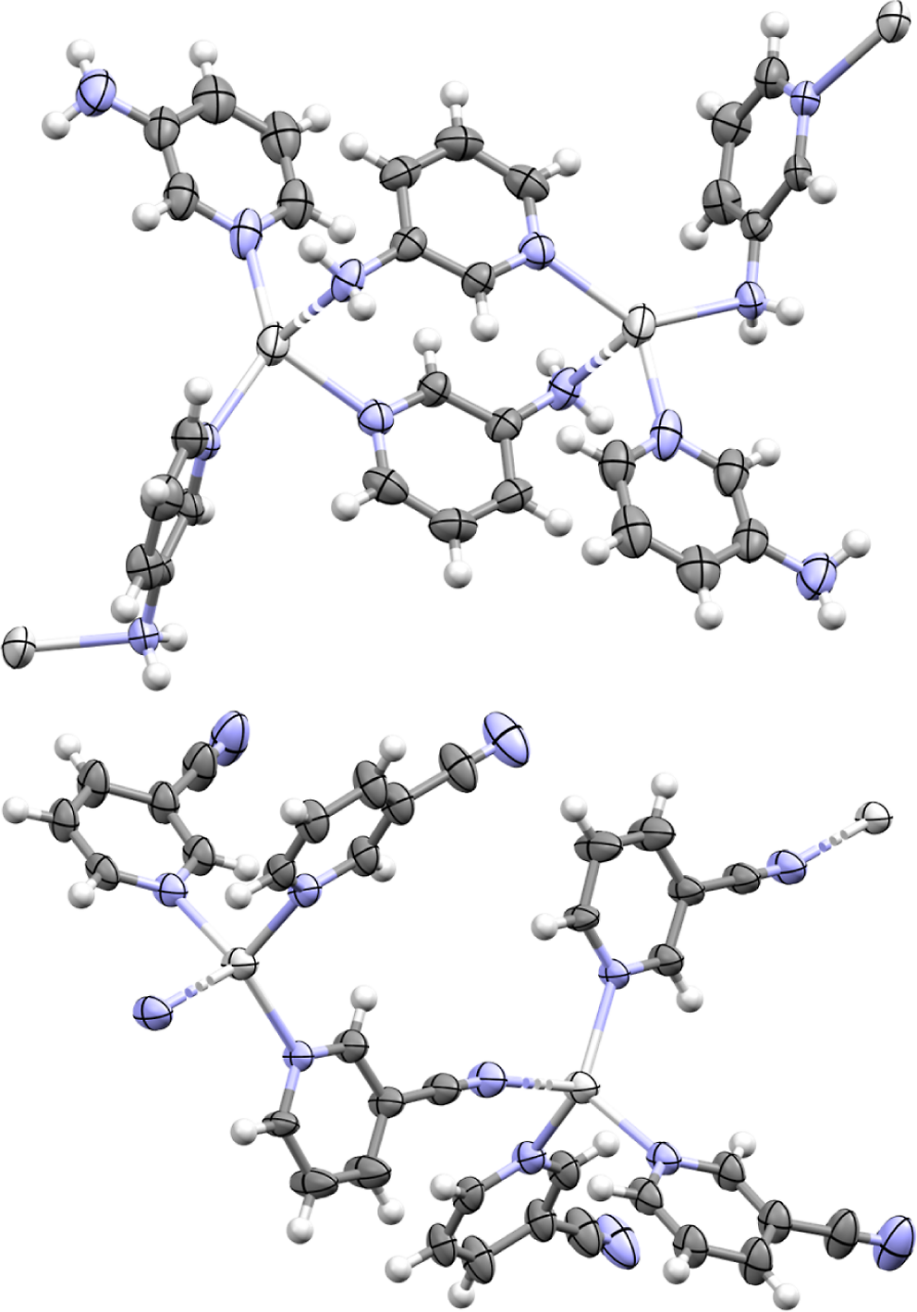
Crystal structures of the 4-coordinate silver(I) complexes **3a·3** (top) and **5a·5** (bottom) showing their 1D polymeric natures (thermal ellipsoids at 50% probability; PF_6_ anions and minor disordered atom positions are omitted for clarity).

Interestingly, the solid-state structures for the potentially coordinating 3-substituents in **3** (NH_2_) and **5** (CN), both returned silver(I) complexes with opportunistically expanded coordination spheres (**3a·3** and **5a·5**, respectively), with the presence of an additional molecule of their respective ligands (**3** or **5**) bridging two tetrahedral silver(I) centers to give 1D polymers (Figure 2) with overall ratios of ligand/silver(I) of 2.5:1 (**3a·3**) and 3:1 (**5a·5**). These tetrahedral coordination spheres were reproducible from evaporation of an initial 2:1 stoichiometry of the free ligands **3** or **5** with AgPF_6_, despite being performed in the weakly coordinating MeCN solvent. The MeCN solvent might have aided in satisfying the valency of the silver(I) centers as reported for **1a**,^32^ though if present, MeCN was ultimately out-competed in favor of the proximal 3-substituted functional groups, NH_2_ and CN.

**Figure 3:**
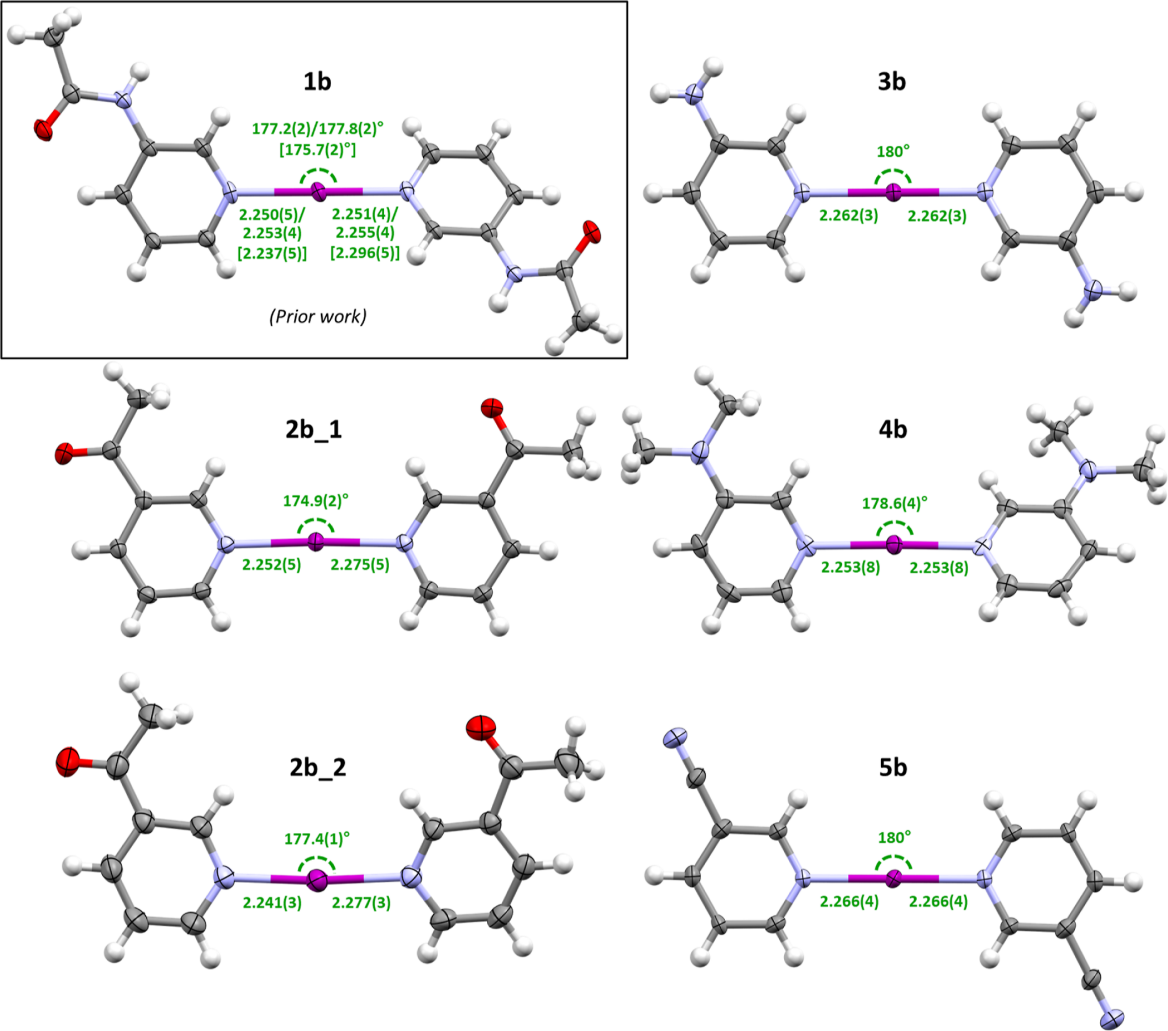
Crystal structures of iodine(I) complexes **2b**–**5b** reported herein and the literature complex **1b** for comparison (structure from crystallization at 298 K shown, with values from crystallization at 253 K given in square brackets),^32^ annotated with their I–N bond lengths (Å) and N–I–N bond angles (thermal ellipsoids at 50% probability; PF_6_ anions and minor disordered atom positions are omitted for clarity).

**Figure 4:**
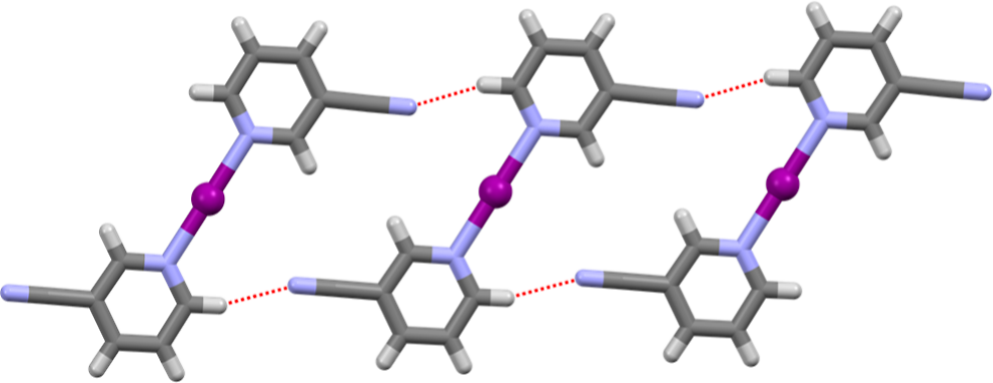
Packing of three [I(3-CNpy)_2_]^+^ cations of **5b** showing the coordination of the 3-CN groups to the hydrogen atom at the 6-position (ortho) of a neighboring iodine(I) cation (represented by a dashed red line; intermolecular distances: (C)N···H(C) = 2.37 Å and (C)N···(H)C = 3.279(7) Å; PF_6_ anions and minor disordered atom positions are omitted for clarity).

The iodine(I) complexes (**2b**–**5b**; Figure 3) did not display the same variety of coordination modes as the silver(I) complexes, owing to the linear coordination geometry of the [L–X–L]^+^ 3-center-4-electron (**3c–4e**) bond, with **2b**–**5b** all being observed as discrete salts, with two crystallographic polymorphs being observed for **2b** (**2b_1** and **2b_2**). It should be noted that two polymorphs were also observed for **1b**, though these occurred from different crystallization temperatures (253 or 298 K).^32^ No intermolecular interactions were found for any of the iodine(I) complexes, except for hydrogen bonding of the NH_2_ group in **3b** with the PF_6_ counterion, and a close contact of the cyano groups of **5b** with the I^+^ center (3.64(1) Å) which is approaching the sum of van der Waals radii (3.53 Å). However, this appears to be a consequence of the packing with all cyano groups coordinating to the H6 (*ortho*-position) atom of the 3-CNpy ligand of a neighboring [I(3-CNpy)_2_]^+^ cation of **5b** in a 1D-array (Figure 4). In general, the iodine(I) complexes showed good adherence to linearity, ranging from perfect, symmetry-defined (180°) linear geometries (**3b** and **5b**) to minor deviations from linearity of 178.6(4)° (**4b**), 177.4(1)° (**2b_2**), or 174.9(2)° (**2b_1**). The relative configurations of the 3-substituents of the iodine(I) complexes (**2b**–**5b**; Figure 3) in the solid state did not appear to have any correlation with **2b_1**, **2b_2**, and **4b** having *syn* configurations and **3b** and **5b***anti* configurations, with **1b** demonstrating both the *anti* configuration when crystallized at 298 K and the *syn* configuration when crystallized at 253 K. The range of I–N bond distances (2.241(3)–2.277(3) Å) for **2b**–**5b** all comfortably fell within the range of values previously reported for known iodine(I) complexes (2.23(1)–2.32(1) Å),^16^ the majority of which incorporated 4-substituted pyridine derivatives,^19^ with the shortest and longest distances both from **2b_2**. The narrow range is unsurprising given the diminished influence of the identity of the substituents due to their 3-positions being resonance-disfavored toward nitrogen coordination, unlike their 4-substituted counterparts. However, given that only seven solid-state examples (from five different species) of iodine(I) complexes incorporating 3-substituted pyridines are known (not including derivatives of isoquinoline),^48^ including the five reported herein for **2b**–**5b**, this cannot be analyzed in more detail.

In comparison to the I–N bond lengths of the two polymorphs reported for **1b** (*cf.***1b_1** = 2.237(5)/2.296(5) Å; **1b_2** = 2.250(5)/2.251(4) Å and 2.253(4)/2.255(4) Å, two crystallographically independent molecules present in the asymmetric unit cell of **1b_2**),^32^ the I–N bond lengths of **2b–5b** are again very similar to each other, many even being crystallographically indistinguishable to a 3σ tolerance, except for the outlying distance of 2.296(5) Å in **1b_1**, which deviates significantly from the I–N bond lengths of all six other solid-state structures. It is unclear if this anomalous I–N bond length has any relevance to the differing reactivity observed for **1b** in comparison to **2b**–**5b**, though given the values of the other iodine(I) complexes, it is likely just a packing effect that will not persist in solution and therefore will have no bearing on the reactivity of **1b** in solution.

While a (relatively) close contact I^+^···I^+^ of 3.777(2) Å was observed in the solid-state structure of [I(py)(4-NMe_2_py)_2_]PF_6_ (py = pyridine)^29^ and of 3.887(1) Å for a helical *bis*-iodine(I) complex,^10^ both of which are shorter than the sum of van der Waals radii of 3.96 Å, the closest I^+^···I^+^ intermolecular distance of **2b**–**5b** observed herein was for **2b_1** (4.3856(5) Å), though it greatly exceeded the sum of van der Waals radii for this potential close contact.

As previously noted for the NMR studies, the 4-substituted analogues of **3b** (with different anions other than PF_6_), **4b**, and **5b** are known, as are their solid-state structures,^24,29,47^ which provide an excellent basis of comparison to structurally assess the impact of structural isomerism (3- vs 4-substitution) on the halogen bonding of the iodine(I) complexes. The halogen bonding of halogen(I) complexes has predominantly been observed to prioritize itself over other electronic structure considerations, which gives rise to the narrow range of solid-state I–N bond lengths despite a wide variety of ligands being utilized in these endeavors. Nevertheless, 4-substituted pyridines like 4-NH_2_py still impart an electronic structure influence to the halogen bonding motif they are incorporated into, as demonstrated by [I(4-NH_2_py)_2_]^+^ possessing one of the shortest reported I–N halogen bonds.^47^

A comparison of the solid-state structures of **3b** with [I(4-NH_2_py)_2_]Cl shows that the I–N bond lengths of **3b** are longer (2.262(3) Å vs 2.240(3)–2.254(2) Å; two independent molecules present in the asymmetric unit cell), though ultimately overlapping when the error of the measurement is accounted for. A similar trend is observed for **4b** (2.253(8) Å) with [I(4-NMe_2_py)_2_]PF_6_ (2.236(3)/2.251(3) Å), as well as for **5b** (2.266(4) Å) and [I(4-CNpy)_2_]PF_6_ (2.241(4)–2.284(4) Å; three independent molecules present in the asymmetric unit cell). This solid-state data suggests that the stability of iodine(I) complexes incorporating 3-substituted pyridines should be comparable to those observed for the 4-substituted analogues, at least in the solid state. However, the longevity enjoyed by [I(4-NMe_2_py)_2_]PF_6_,^24^ which can persist for months at ambient temperature, is not mimicked by **4b** or any other of the 3-substituted pyridine iodine(I) complexes. In strong contrast to their 4-substituted pyridine analogues, the iodine(I) complexes **2b**–**5b** were all observed to degrade within days if not kept at reduced temperatures, for example, a freshly prepared sample of **2b** (white solid) was observed to completely decompose to a dark brown solid within hours at ambient temperature.

The formation of the reaction product **1c**, which entailed the breaking and iodination of a normally inert ^t^BuOMe upon reaction with iodine(I) complex **1b** was the first time such reactivity had been observed for an iodine(I) complex. However, when viewed from the broader perspective of being a Lewis acid, the reactivity of the iodine(I) complex **1b** toward ethers can be seen as analogous to that reported for other Lewis and Brönsted acids,^49,50^ as can the subsequent alkylation of the liberated pyridine-based nucleophile **1**. The observation of brominated products from the cleavage of ethers was also noted upon reaction with Br_2_ when strongly acidic conditions were employed.^50^

The reaction of **1b** was found to proceed over several days with a huge excess of ^t^BuOMe, was reliably reproducible, and theorized to originate from **1b** acting as a source of “I^+^” to generate, in situ, the highly reactive IOMe and ^t^Bu^+^ (itself subsequently losing a hydrogen atom to generate 2-methylpropene), instigating the reaction. Extensive ^1^H NMR studies, attempting to monitor the progression of the stoichiometric reaction of **1b** and ^t^BuOMe in CD_3_CN unfortunately failed to proceed after 72 h. This suggested that the formation of **1c** required the enormous excess of ^t^BuOMe to be present to occur in appreciable amounts, which rendered such monitoring ^1^H NMR studies no longer fit for purpose.

**Figure 5:**
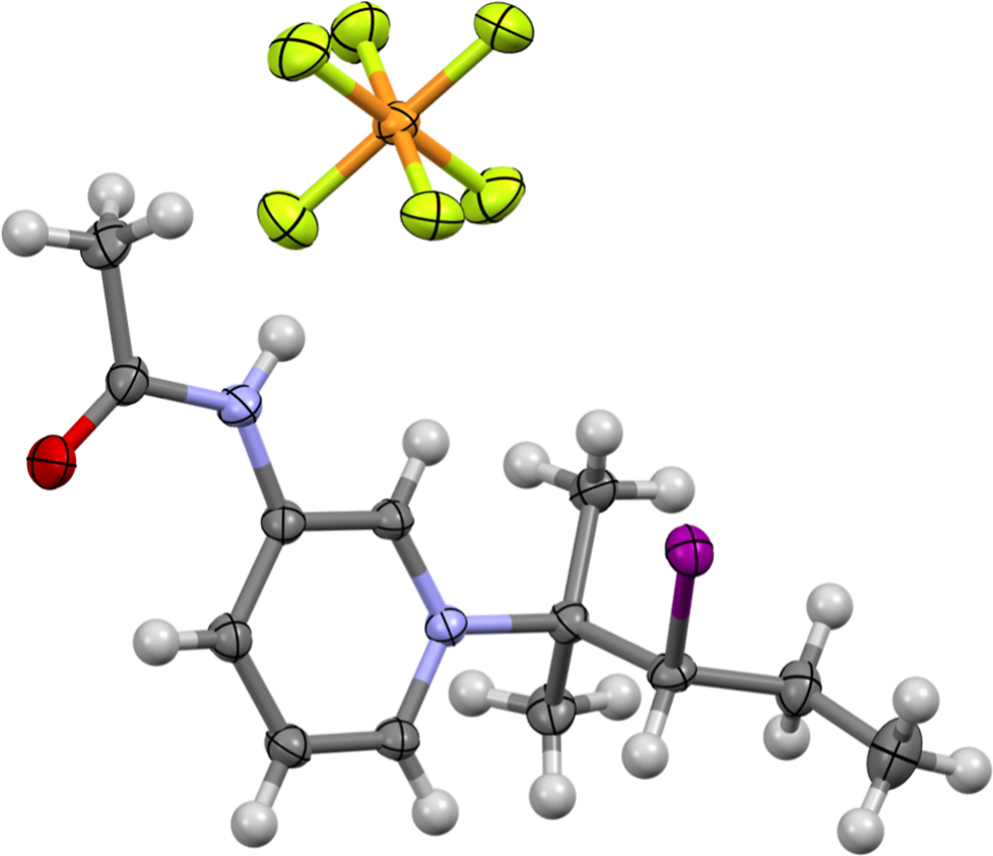
Crystal structure of complex **1d**, formed upon crystallization of iodine(I) complex **1b** in the presence of a large excess of ^i^Pr_2_O (thermal ellipsoids at 50% probability).

Nevertheless, the scope of the reactivity could still be explored by reproducing the reaction conditions for the synthesis of **1c** using other common ethers, with the common laboratory anti-solvents Et_2_O and ^i^Pr_2_O selected for this purpose. For these solvents, the previously proposed mechanism would therefore generate the hypoiodites IOEt and IO^i^Pr, which was reasonable, however, the accompanying carbocation intermediates of Et^+^ and ^i^Pr^+^ would be less stabilized than the tertiary ^t^Bu^+^ due to the decreasing stabilization of the positive inductive effect (^t^Bu^+^ > ^i^Pr^+^ ≫ Et^+^), with the expectation of the primary carbocation Et^+^ (and possibly the secondary carbocation ^i^Pr^+^) being too transient for the novel reactivity to be observed here. The probable formation of ^i^Pr^+^, and subsequent conversion to propene via the loss of a hydrogen atom, was demonstrated by the observation of the analogous product **1d** (Figure 5) being isolated from the reaction of **1b** and ^i^Pr_2_O, though no similar species was observed upon reaction of **1b** with Et_2_O.

There is precedence for the stabilization of carbocations with a pair of Lewis bases in a directly analogous fashion to *bis*(pyridine)iodine(I) complexes, which may play a role in the mechanism.^51^ Additionally, the synthesis of analogous N-alkylated-β-halogenated compounds upon reaction of aromatic amines and alkenes has been reported.^52−54^ A tentative mechanism could therefore be suggested from the initial formation of 2-methyl-2-pentene from propene (itself from ^i^Pr^+^) that in turn reacts with a liberated 3-acetaminopyridine ligand and a source of I^+^ (either the iodine(I) complex **1b** or the *in situ* generated IO^i^Pr) to give **1d**. This is supported by the knowledge that 2-methyl-2-pentene can be directly formed from ^i^PrOH,^55^ with ^i^PrOI also being a potential chemical precursor. Nevertheless, the exact reaction mechanism for the formation of **1d** remains unclear, though it is clear that it involves the addition of two *in situ* generated propyl fragments and an iodine atom, making it a potential method for creating synthetically useful C–C and C–I bonds in the same vein as the ubiquitous Barluenga’s reagent.

With respect to the breadth of this unexpected reactivity among other structurally related 3-substituted iodine(I) complexes, no analogous reactivity as that observed for **1b** with ^t^BuOMe (**1c**) or ^i^Pr_2_O (**1d**) was observed for any of the other iodine(I) complexes synthesized herein (**2b–5b**), indicating that it is likely several properties of **1b** that contribute to its reactivity, such as the 3-substitution of the acetamino group, it being simultaneously a hydrogen bond donor (NH) and acceptor (C=O), or a consequence of it being a hierarchical material.

## Conclusions

Iodine(I) complexes (**2b**–**5b**), synthesized via cation exchange from their respective silver(I) complexes (**2a**–**5a**), incorporating functionally related substituents to that of the previously reported **1b** (including 3-Acpy, 3-NH_2_py, and 3-NMe_2_py, as well as 3-CNpy to test the boundaries of iodine(I) formation), were synthesized and studied in both solution (with the exception of the highly reactive **5b**) and the solid state for all complexes reported herein. The solid-state structures of **1b**–**5b** represented the only five examples of iodine(I) complexes bearing 3-substituted pyridines (encompassing seven solid-state structures due to **1b** and **2b** both having two polymorphs), and internal comparisons indicated that these species demonstrated the same consistency observed for all reported iodine(I) complexes in their I–N bond lengths (and N–I–N angles, as expected for halogen bonding originating from a pair of p-hole type interactions),^15,56^ with a particularly narrow range of I–N bond lengths of only 0.04 Å (compared to the 0.09 Å of all currently reported iodine(I) complexes). The ^1^H–^15^N HMBC determined ^15^N NMR chemical shifts demonstrated the same general trend of being more shielded going from uncoordinated ligand to silver(I) to iodine(I) complex as previously observed for analogous iodine(I) complexes, though significantly deshielded in comparison to their iodine(I) complexes bearing 4-substituted pyridines, commensurate with the electronic environments of the nitrogen atoms in the 3-substituted pyridines utilized herein. None of the new iodine(I) complexes (**2b**–**5b**) demonstrated the same unexpected reactivity as **1b** with ^t^BuOMe to give **1c**. However, the reactivity of **1b** was further extended to a second etheric solvent (^i^Pr_2_O) to give the N-alkylated salt **1d**, which (similar to **1c**) included C–I bond formation but also involved synthetically useful C–C bond formation as well, revealing further potential of this serendipitous reactivity if its origins can be fully established.

## Experimental Section

### General Considerations

All reagents and solvents were obtained from commercial suppliers and used without further purification. The NMR and solid-state data for **1**, **1a**, **1b**, and **1c** have been previously reported.^32^ For structural NMR assignments, ^1^H NMR and ^1^H–^15^N NMR correlation spectra were recorded on a Bruker AVANCE III 500 MHz spectrometer at 25 °C in CD_2_Cl_2_ or CD_3_CN. Chemical shifts are reported on the δ scale in ppm using the residual solvent signal as internal standard (CH_2_Cl_2_ in CD_2_Cl_2_: δ_H_ 5.32; CH_3_CN in CD_3_CN: δ_H_ 1.94) or for ^1^H–^15^N NMR spectroscopy to an external CD_3_NO_2_ standard. For ^1^H NMR spectroscopy, each resonance was assigned according to the following conventions: chemical shift (δ) measured in ppm, observed multiplicity, observed coupling constant (*J* Hz), and number of hydrogens. Multiplicities are denoted as: s (singlet), d (doublet), t (triplet), q (quartet), m (multiplet), and br (broad). For the ^1^H–^15^N HMBC spectroscopy, spectral windows of 4–8 ppm (^1^H) and 300 or 400 ppm (^15^N) were used, with 1024 points in the direct dimension and 512 increments used in the indirect dimension, with subsequent peak shape analyses being performed to give the reported ^15^N NMR resonances.

The single-crystal X-ray data for **1d**, **1e·1**, **4b**, and **4f** were collected at 120 K using an Agilent SuperNova dual wavelength diffractometer with an Atlas detector using mirror-monochromated Cu Kα (λ = 1.54184 Å) or Mo Kα (λ = 0.71073 Å) radiation. The single-crystal X-ray data for **2a**, **3a·3**, **4a**, **5a·5**, and **5b** were collected at 120 K using a Rigaku XtaLAB Synergy-R diffractometer with a HyPix-Arc 100 detector using mirror-monochromated Cu Kα (λ = 1.54184 Å) radiation. The single-crystal X-ray data for **3b** was collected at 120 K using an Agilent SuperNova dual wavelength diffractometer with a HyPix-Arc 100 detector using mirror-monochromated Cu Kα (λ = 1.54184 Å). The single-crystal X-ray data for **2b_2** was collected at 120 K using an Agilent SuperNova diffractometer with an Eos detector using mirror-monochromated Mo Kα (λ = 0.71073 Å) radiation. The single-crystal X-ray data for **2b_1** was collected at 170 K using a Bruker-Nonius Kappa CCD diffractometer with an APEX-II detector with graphite-monochromatized Mo Kα (λ = 0.71073 Å) radiation, with the COLLECT program for data collection and DENZO/SCALEPACK for the data reduction.^57,58^ All structures were solved by intrinsic phasing (SHELXT)^59^ and refined by full-matrix least squares on *F*^2^ using Olex2,^60^ utilizing the SHELXL module.^61^ Anisotropic displacement parameters were assigned to non-H atoms, and isotropic displacement parameters for all H atoms were constrained to multiples of the equivalent displacement parameters of their parent atoms with U_iso_(H) = 1.2 U_eq_(NH_2_, NH, aromatic, methylene, methine) or U_iso_(H) = 1.5 U_eq_(methyl) of their respective parent atoms.

Please refer to the Supporting Information for details of the synthesis and characterization of all complexes described herein.

## Supporting Information Available

Additional experimental and characterization details, crystallographic details, ^15^N NMR comparison data, and NMR spectra (PDF)
Crystal structures of reported compounds (CIF)

## Supplementary Material

ao3c03097_si_001.pdf

ao3c03097_si_002.cif

## Abbreviations

3-AcNHpy: 3-acetaminopyridine
3-Acpy: 3-acetylpyridine
3-NH_2_py: 3-aminopyridine
3-NMe_2_py: 3-dimethylaminopyridine
DCM: dichloromethane
DIPE: diisopropyl ether
TBME: *tert*-butylmethyl ether
