# Supplementary material for: Iodine(I) and Silver(I) Complexes Incorporating 3-Substituted Pyridines
Source: ACS Omega. 2023 Jun 21;8(26):24064–71. doi: 10.1021/acsomega.3c03097 (PMC10324066; doi:10.1021/acsomega.3c03097)
Supplement: Supplementary file 1 — ao3c03097_si_001.pdf [file ao3c03097_si_001.pdf]

Supporting Information

Iodine(I) and Silver(I) Complexes Incorporating 3-substituted Pyridines

Kari Rissanen[a*] and Jas S. Ward[a*]

[a] University of Jyvaskyla, Department of Chemistry, Jyväskylä 40014, Finland.

E-mail: kari.t.rissanen@jyu.fi, james.s.ward@jyu.fi

Contents

Synthesis and Characterisation S2

General Considerations S2

Nomenclature S3

Free Ligands S4

Silver(I) Complexes S5

Iodine(I) Complexes S8

Reaction Products of **1b** S13

Comparison Tables of ^{15}N NMR Chemical Shifts S15

NMR Spectra S16

References S43

Synthesis and Characterisation

General Considerations

All reagents and solvents were obtained from commercial suppliers and used without further purification. The NMR and solid-state data for **1**, **1a**, **1b**, and **1c** have been previously reported.[1] For structural NMR assignments, ^{1}H NMR and ^{1}H-^{15}N NMR correlation spectra were recorded on a Bruker Avance III 500 MHz spectrometer at 25°C in CD_2Cl_2 or CD_3CN. Chemical shifts are reported on the δ scale in ppm using the residual solvent signal as internal standard (CH_2Cl_2 in CD_2Cl_2: δ_H 5.32; CH_3CN in CD_3CN: δ_H 1.94), or for ^{1}H-^{15}N NMR spectroscopy, to an external CD_3NO_2 standard. For the ^{1}H NMR spectroscopy, each resonance was assigned according to the following conventions: chemical shift (δ) measured in ppm, observed multiplicity, observed coupling constant (*J* Hz), and number of hydrogens. Multiplicities are denoted as: s (singlet), d (doublet), t (triplet), q (quartet), m (multiplet), and br (broad). For the ^{1}H-^{15}N HMBC spectroscopy, spectral windows of 4-8 ppm (^{1}H) and 300 or 400 ppm (^{15}N) were used, with 1024 points in the direct dimension and 512 increments used in the indirect dimension, with subsequent peak shape analysis being performed to give the reported ^{15}N NMR resonances.

The single crystal X-ray data for **1d**, **1e·1**, **4b**, and **4f** were collected at 120 K using an Agilent SuperNova dual wavelength diffractometer with an Atlas detector using mirror-monochromated Cu-Kα (λ = 1.54184 Å) or Mo-Kα (λ = 0.71073 Å) radiation. The single crystal X-ray data for **2a**, **3a·3**, **4a**, **5a·5**, and **5b** were collected at 120 K using a Rigaku XtaLAB Synergy-R diffractometer with a HyPix-Arc 100 detector using mirror-monochromated Cu-Kα (λ = 1.54184 Å) radiation. The single crystal X-ray data for **3b** was collected at 120 K using an Agilent SuperNova dual wavelength diffractometer with a HyPix-Arc 100 detector using mirror-monochromated Cu-Kα (λ = 1.54184 Å). The single crystal X-ray data for **2b_2** was collected at 120 K using an Agilent SuperNova diffractometer with an Eos detector using mirror-monochromated Mo-Kα (λ = 0.71073 Å) radiation. The single crystal X-ray data for **2b_1** was collected at 170 K using Bruker-Nonius Kappa CCD diffractometer with an APEX-II detector with graphite-monochromatised Mo-Kα (λ = 0.71073 Å) radiation, with the COLLECT program for data collection and DENZO/SCALEPACK for the data reduction.[2,3] All structures were solved by intrinsic phasing (SHELXT)[4] and refined by full-matrix least squares on F^2 using Olex2,[5] utilising the SHELXL module.[6] Anisotropic displacement parameters were assigned to non-H atoms and isotropic displacement parameters for all H atoms were constrained to multiples of the equivalent displacement parameters of their parent atoms with U_{iso}(H) = 1.2 U_{eq}(NH_2, NH, aromatic, methylene, methine) or U_{iso}(H) = 1.5 U_{eq}(methyl) of their respective parent atoms. The X-ray single crystal data and CCDC numbers (2253721-2253732) of all new structures are included below.

The following abbreviations are used: 3-AcNHpy = 3-acetaminopyridine, 3-Acpy = 3-acetylpyridine, 3-NH_2py = 3-aminopyridine, 3-NMe_2py = 3-dimethylaminopyridine, DCM = dichloromethane, DIPE = diisopropyl ether, TBME = ^{t}butylmethyl ether.

Nomenclature

**Compound/ Complex**	**Free Ligand**	**Silver(I)**	**Iodine(I)**
3-AcNHpy	**1**[1]	**1a**[1]	**1b**[1]
3-Acpy	**2**	**2a**	**2b**
3-NH_2py	**3**	**3a**	**3b**
3-NMe_2py	**4**	**4a**	**4b**
3-CNpy	**5**	**5a**	**5b**

**Reaction Products of 1b**	
With TBME	**1c**[1]
With DIPE	**1d**

**Side Products**	
**H[PF_6] Salt**	**1e**
**Hydronium**	**4f**

Free Ligands

3-acetylpyridine (**3-Acpy; 2**):

^{1}H NMR (500 MHz, CD_3CN) δ 9.11 (s, 1H), 8.74 (d, *J* = 2.8 Hz, 1H), 8.23 (br.dd, *J* = 4.2, 1.8 Hz, 1H), 7.46 (dd, *J* = 7.6, 4.0 Hz, 1H), 2.59 (s, 3H); ^{15}N NMR (HMBC, CD_3CN) δ -65.3.

^{1}H NMR (500 MHz, CD_2Cl_2) δ 9.13 (s, 1H), 8.75 (d, *J* = 4.4 Hz, 1H), 8.20 (d, *J* = 7.9 Hz, 1H), 7.42 (dd, *J* = 7.9, 4.8 Hz, 1H), 2.61 (s, 3H); ^{15}N NMR (HMBC, CD_2Cl_2) δ -66.4.

3-aminopyridine (**3-NH_2py; 3**):

^{1}H NMR (500 MHz, CD_3CN) δ 8.00 (d, *J* = 2.4 Hz, 1H), 7.85 (dd, *J* = 4.5, 1.1 Hz, 1H), 7.03 (dd, *J* = 8.0, 4.5 Hz, 1H), 6.96 (ddd, *J* = 8.2, 2.7, 1.4 Hz, 1H), 4.25 (br.s, 2H); ^{15}N NMR (HMBC, CD_3CN) δ -64.0 (pyridinic), -330.8 (NH_2).

3-dimethylaminopyridine (**3-NMe_2py; 4**):

^{1}H NMR (500 MHz, CD_3CN) δ 8.11 (d, *J* = 3.0 Hz, 1H), 7.88 (d, *J* = 4.5 Hz, 1H), 7.13 (dd, *J* = 8.5, 4.5 Hz, 1H), 7.05 (dd, *J* = 8.4, 2.9 Hz, 1H), 2.93 (s, 6H); ^{15}N NMR (HMBC, CD_3CN) δ -65.3 (pyridinic), -339.4 (NMe_2).

^{1}H NMR (500 MHz, CD_2Cl_2) δ 8.14 – 8.09 (m, 1H), 7.94 – 7.89 (m, 1H), 7.11 (dddd, *J* = 8.5, 4.6, 1.8, 0.5 Hz, 1H), 6.98 (ddt, *J* = 8.5, 3.1, 1.5 Hz, 1H), 2.95 (s, 6H); ^{15}N NMR (HMBC, CD_2Cl_2) δ -67.4 (pyridinic), -339.3 (NMe_2).

3-cyanopyridine (**3-CNpy; 5**):

^{1}H NMR (500 MHz, CD_3CN) δ 8.89 (d, *J* = 1.3 Hz, 1H), 8.80 (dd, *J* = 4.9, 1.5 Hz, 1H), 8.09 (dt, *J* = 8.0, 1.9 Hz, 1H), 7.51 (ddd, *J* = 8.0, 5.0, 0.8 Hz, 1H); ^{15}N NMR (HMBC, CD_3CN) δ -63.2.

^{1}H NMR (500 MHz, CD_2Cl_2) δ 8.88 (d, *J* = 1.4 Hz, 1H), 8.80 (dd, *J* = 4.9, 1.7 Hz, 1H), 7.98 (dt, *J* = 8.0, 1.9 Hz, 1H), 7.44 (ddd, *J* = 8.0, 4.9, 0.9 Hz, 1H); ^{15}N NMR (HMBC, CD_2Cl_2) δ -63.5.

Silver(I) Complexes

The silver(I) complexes could all be prepared in quantitative yields by the simple addition of 2 equivalents of the respective ligands (**2**-**5**; 0.12 mmol) with 1 equivalent of $AgPF_6$ (15.2 mg, 0.06 mmol) in MeCN (4 mL).

[Ag(3-Acpy)$_2$]PF$_6$ (**2a**):

^{1}H NMR (500 MHz, CD_3CN) δ 9.08 (d, *J* = 1.5 Hz, 2H), 8.72 (dd, *J* = 4.9, 1.5 Hz, 2H), 8.35 – 8.29 (m, 2H), 7.56 (ddd, *J* = 8.0, 5.0, 0.6 Hz, 2H), 2.60 (s, 6H); ^{15}N NMR (HMBC, CD_3CN) δ -83.6.

Crystals suitable for single crystal X-ray diffraction were obtained by evaporation of a MeCN solution of **2a**. Crystal data for **2a**: CCDC-2253723, $[C_{14}H_{14}AgN_2O_2]PF_6$, M = 495.11, colourless plate, 0.01 × 0.07 × 0.12 mm, orthorhombic, space group *Pccn*, a = 12.4247(3) Å, b = 13.1772(3) Å, c = 20.1856(4) Å, V = 3304.84(13) Å^3, Z = 8, D_{calc} = 1.990 gcm^{-3}, F(000) = 1952, μ = 11.45 mm^{-1}, T = 120.0(1) K, θ_{max} = 74.5°, 3388 total reflections, 3155 with $I_o > 2\sigma(I_o)$, R_{int} = 0.036, 3388 data, 262 parameters, 45 restraints, GooF = 1.08, 1.31 < dΔρ < -1.07 eÅ^{-3}, $R[F^2 > 2\sigma(F^2)]$ = 0.047, $wR(F^2)$ = 0.128.

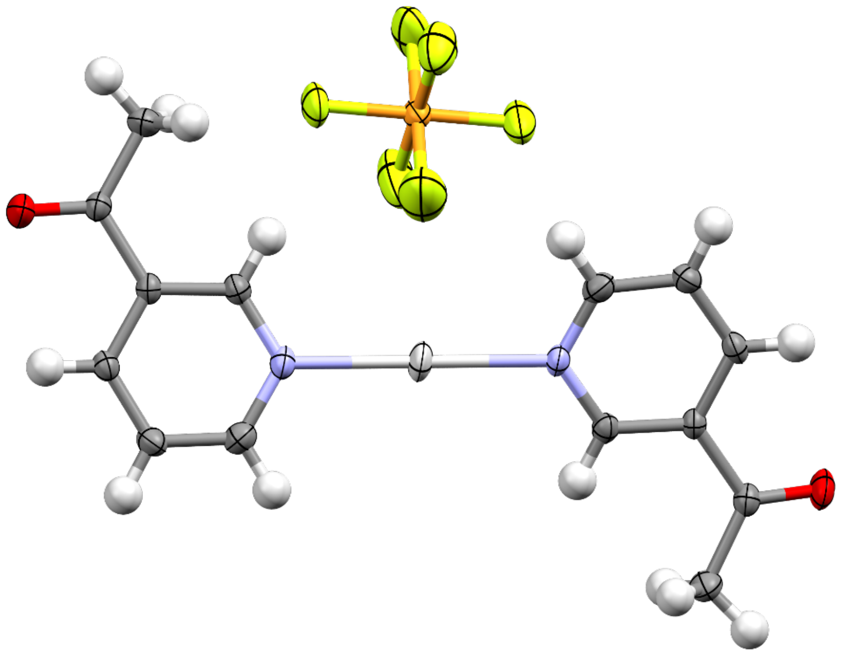

Figure S1: The crystal structure of **2a**. Colour key: light grey = silver, orange = phosphorus, lime green = fluorine, red = oxygen, blue = nitrogen, dark grey = carbon, white = hydrogen.

[Ag(3-NH$_2$py)$_2$]PF$_6$ (**3a**):

^{1}H NMR (500 MHz, CD_3CN) δ 7.95 (d, *J* = 2.5 Hz, 2H), 7.80 (dd, *J* = 4.8, 1.2 Hz, 2H), 7.17 (dd, *J* = 8.3, 4.8 Hz, 2H), 7.11 (ddd, *J* = 8.3, 2.6, 1.4 Hz, 2H), 4.48 (s, 4H); ^{15}N NMR (HMBC, CD_3CN) δ -98.8 (pyridinic), -329.0 (NH_2).

Crystals suitable for single crystal X-ray diffraction were obtained by evaporation of a MeCN solution of **3a**. Crystal data for **3a·3**: CCDC-2253726, $([C_{25}H_{30}Ag_2N_{10}]PF_6)_n$, M = 976.27, colourless plate, 0.01 × 0.06 × 0.11 mm, monoclinic, space group *C*2/*c*, a = 14.7593(6) Å, b = 13.9951(6) Å, c = 16.7787(8) Å, β = 102.335(4)°, V = 3385.8(3) Å^3, Z = 4, D_{calc}

= 1.915 gcm^{-3}, F(000) = 1928, μ = 11.12 mm^{-1}, T = 120.0(1) K, θ_{max} = 74.5°, 3467 total reflections, 2896 with $I_o > 2\sigma(I_o)$, R_{int} = 0.070, 3467 data, 269 parameters, no restraints, GooF = 1.04, 0.95 < dΔρ < -0.97 eÅ^{-3}, $R[F^2 > 2\sigma(F^2)]$ = 0.058, $wR(F^2)$ = 0.145.

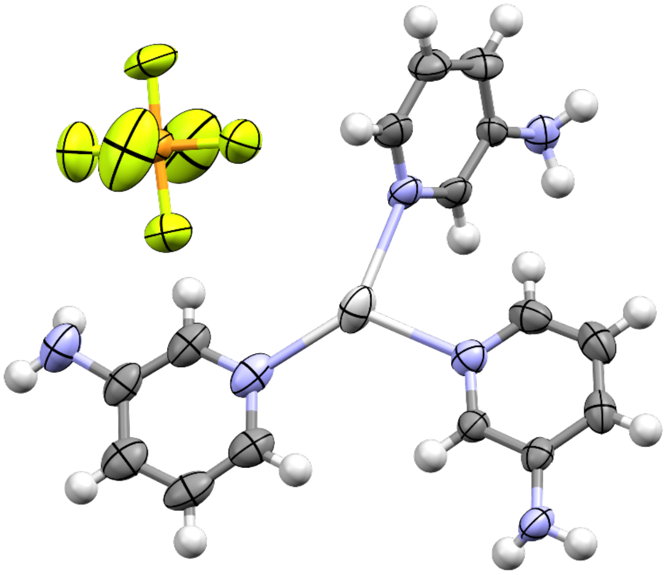

Figure S2: The asymmetric unit cell of **3a·3**. Colour key: light grey = silver, orange = phosphorus, lime green = fluorine, blue = nitrogen, dark grey = carbon, white = hydrogen.

[Ag(3-NMe$_2$py)$_2$]PF$_6$ (**4a**):

^{1}H NMR (500 MHz, CD_3CN) δ 8.03 (d, *J* = 3.0 Hz, 2H), 7.85 (dd, *J* = 4.8, 1.1 Hz, 2H), 7.30 (dd, *J* = 8.7, 4.8 Hz, 2H), 7.22 (ddd, *J* = 8.7, 3.1, 1.2 Hz, 2H), 2.97 (s, 12H); ^{15}N NMR (HMBC, CD_3CN) δ -103.3 (pyridinic), -335.7 (NMe$_2$).

^{1}H NMR (500 MHz, CD_2Cl_2) δ 8.01 (d, *J* = 3.0 Hz, 2H), 7.86 (dd, *J* = 5.0, 0.9 Hz, 2H), 7.40 (dd, *J* = 8.8, 5.0 Hz, 2H), 7.25 (ddd, *J* = 8.8, 3.0, 0.8 Hz, 2H), 3.04 (s, 12H); ^{15}N NMR (HMBC, CD_2Cl_2) δ -132.3 (pyridinic), -332.7 (NMe$_2$).

Crystals suitable for single crystal X-ray diffraction were obtained from a DCM solution of **4a** vapour diffused with DIPE. Crystal data for **4a**: CCDC-2253728, [$C_{14}H_{20}AgN_4$]PF_6, M = 497.18, colourless plate, 0.04 × 0.04 × 0.09 mm, triclinic, space group *P*-1 (No. 2), a = 8.4979(2) Å, b = 10.0145(3) Å, c = 11.9821(3) Å, α = 94.170(2)°, β = 104.433(2)°, γ = 113.180(3)°, V = 890.93(5) Å^3, Z = 2, D_{calc} = 1.853 gcm^{-3}, F(000) = 496, μ = 10.56 mm^{-1}, T = 120.0(1) K, θ_{max} = 74.5°, 3618 total reflections, 3352 with $I_o > 2\sigma(I_o)$, R_{int} = 0.029, 3618 data, 239 parameters, no restraints, GooF = 1.07, 0.86 < dΔρ < -0.86 eÅ^{-3}, $R[F^2 > 2\sigma(F^2)]$ = 0.027, $wR(F^2)$ = 0.070.

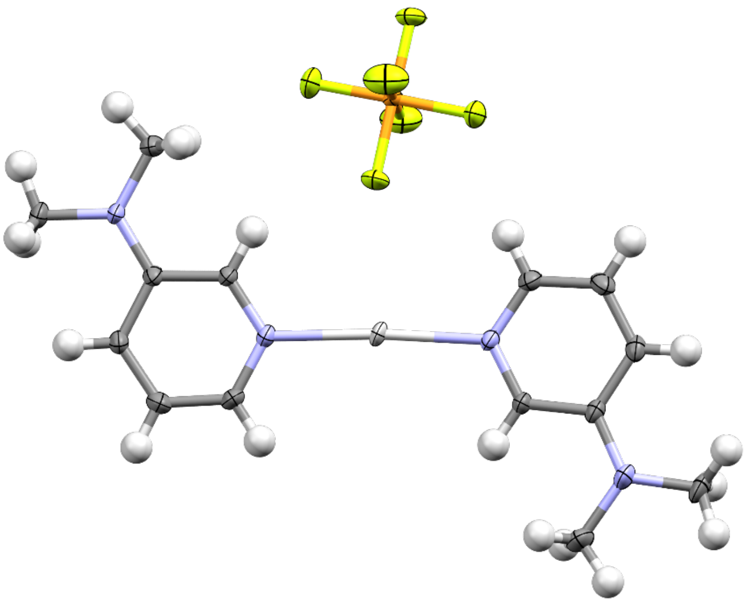

Figure S3: The crystal structure of **4a**. Colour key: light grey = silver, orange = phosphorus, lime green = fluorine, blue = nitrogen, dark grey = carbon, white = hydrogen.

[Ag(3-CNpy)$_2$]PF$_6$ (**5a**):

^{1}H NMR (500 MHz, CD_3CN) δ 8.88 (d, *J* = 1.3 Hz, 2H), 8.79 (dd, *J* = 5.0, 1.5 Hz, 2H), 8.12 (dt, *J* = 8.0, 1.9 Hz, 2H), 7.54 (ddd, *J* = 8.0, 5.0, 0.7 Hz, 2H); ^{15}N NMR (HMBC, CD_3CN) δ -68.8.

Crystals suitable for single crystal X-ray diffraction were obtained by evaporation of a MeCN solution of **5a**. Crystal data for **5a·5**: CCDC-2253731, ([$C_{18}H_{12}AgN_6$]PF_6)$_n$, M = 565.18, colourless plate, 0.01 × 0.05 × 0.08 mm, orthorhombic, space group *Pnma*, a = 16.2603(12) Å, b = 13.7681(17) Å, c = 9.2622(8) Å, V = 2073.6(3) Å^3, Z = 4, D_{calc} = 1.810 gcm^{-3}, F(000) = 1112, μ = 9.21 mm^{-1}, T = 120.0(1) K, θ_{max} = 74.4°, 2194 total reflections, 1745 with $I_o > 2\sigma(I_o)$, R_{int} = 0.057, 2194 data, 166 parameters, no restraints, GooF = 1.09, 0.79 < dΔρ < -1.97 eÅ^{-3}, $R[F^2 > 2\sigma(F^2)]$ = 0.062, $wR(F^2)$ = 0.175.

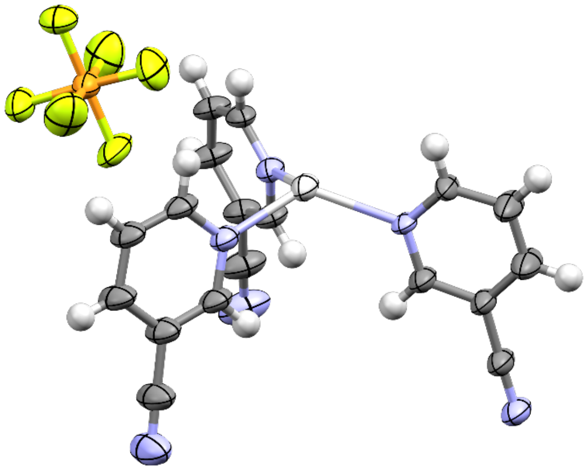

Figure S4: The crystal structure of **5a·5**. Colour key: light grey = silver, orange = phosphorus, lime green = fluorine, blue = nitrogen, dark grey = carbon, white = hydrogen.

Iodine(I) Complexes

All iodine(I) complexes were prepared using the same quantitative general method, which is given below using **2b** as an example. Spectroscopic samples were typically prepared directly using deuterated solvents (CD_3CN or CD_2Cl_2) on a 0.05 mmol scale.

[I(3-Acpy)$_2$]PF$_6$ (**2b**):

To an MeCN (4 mL) solution of **2a** (29.7 mg, 0.06 mmol), elemental iodine (15.2 mg, 0.06 mmol) was added to immediately give a pale pink solution and yellow precipitate (AgI) once all the I_2 had been consumed (<5 minutes), followed by isolation of the filtrate by gravity filtration.

^{1}H NMR (500 MHz, CD_3CN) δ 9.22 (s, 2H), 8.92 (d, J = 4.6 Hz, 2H), 8.68 (d, J = 7.2 Hz, 2H), 7.76 (dd, J = 7.7, 5.8 Hz, 2H), 2.65 (s, 6H); ^{15}N NMR (HMBC, CD_3CN) δ -175.9.

^{1}H NMR (500 MHz, CD_2Cl_2) δ 9.28 (s, 2H), 8.93 (d, J = 5.4 Hz, 2H), 8.68 (d, J = 8.0 Hz, 2H), 7.78 (dd, J = 7.8, 5.7 Hz, 2H), 2.71 (s, 6H); ^{15}N NMR (HMBC, CD_2Cl_2) δ -175.2.

Crystals suitable for single crystal X-ray diffraction were obtained from a DCM solution of **2b** vapour diffused with Et_2O. Crystal data for **2b_1**: CCDC-2253724, $[C_{14}H_{14}IN_2O_2]PF_6$, M = 514.14, colourless needle, 0.08 × 0.14 × 0.38 mm, monoclinic, space group $P2_1/c$, a = 16.5965(6) Å, b = 14.2928(3) Å, c = 7.7195(3) Å, β = 97.238(2)°, V = 1816.55(10) Å^3, Z = 4, D_{calc} = 1.880 gcm^{-3}, F(000) = 1000, μ = 1.92 mm^{-1}, T = 170(1) K, θ_{max} = 27.1°, 3988 total reflections, 3251 with $I_o > 2\sigma(I_o)$, R_{int} = 0.041, 3988 data, 271 parameters, 126 restraints, GooF = 1.05, 0.89 < dΔρ < -0.78 eÅ^{-3}, $R[F^2 > 2\sigma(F^2)]$ = 0.046, $wR(F^2)$ = 0.135.

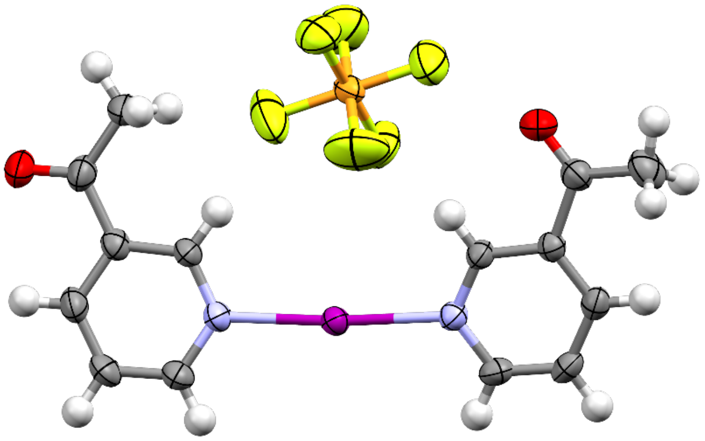

Figure S5: The crystal structure of **2b_1**. Colour key: purple = iodine, orange = phosphorus, lime green = fluorine, red = oxygen, blue = nitrogen, dark grey = carbon, white = hydrogen.

Crystals suitable for single crystal X-ray diffraction were obtained from a DCM solution of **2b** vapour diffused with TBME. Crystal data for **2b_2**: CCDC-2253725, $[C_{14}H_{14}IN_2O_2]PF_6$, M = 514.14, colourless block, 0.10 × 0.14 × 0.14 mm, monoclinic, space group $C2/c$, a = 14.1946(3) Å, b = 11.1506(2) Å, c = 23.0316(4) Å, β = 99.701(2)°, V = 3593.28(12) Å^3, Z = 8, D_{calc} = 1.901 gcm^{-3}, F(000) = 2000, μ = 1.94 mm^{-1}, T = 120.0(1) K, θ_{max} = 26.4°, 3673 total reflections, 3264

with $I_o > 2\sigma(I_o)$, R_{int} = 0.024, 3673 data, 271 parameters, 126 restraints, GooF = 1.04, 1.53 < dΔρ < -0.94 eÅ^{-3}, $R[F^2 > 2\sigma(F^2)]$ = 0.037, $wR(F^2)$ = 0.100.

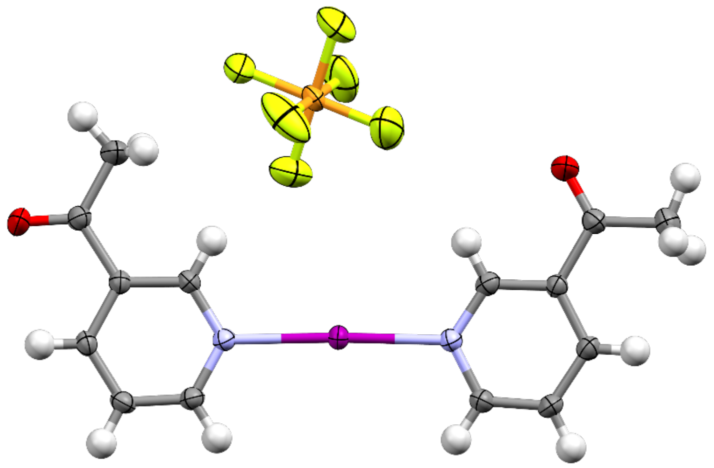

Figure S6: The crystal structure of **2b_2**. Colour key: purple = iodine, orange = phosphorus, lime green = fluorine, red = oxygen, blue = nitrogen, dark grey = carbon, white = hydrogen.

$[I(3\text{-}NH_2py)_2]PF_6$ (**3b**):

^{1}H NMR (500 MHz, CD_3CN) δ 8.07 (s, 2H), 7.97 (d, *J* = 4.7 Hz, 2H), 7.37 (d, *J* = 7.0 Hz, 2H), 7.22 (dd, *J* = 8.2, 5.3 Hz, 2H), 4.91 (br.s, 4H); ^{15}N NMR (HMBC, CD_3CN) δ -173.9 (pyridinic), -323.9 (NH_2).

Crystals suitable for single crystal X-ray diffraction were obtained by evaporation of a concentrated MeCN solution of **3b**. Crystal data for **3b**: CCDC-2253727, $[C_{10}H_{12}IN_4]PF_6$, M = 460.11, colourless block, 0.05 × 0.06 × 0.20 mm, monoclinic, space group *C*2/*m*, a = 13.7973(2) Å, b = 6.50426(10) Å, c = 8.77203(15) Å, β = 106.6644(18)°, V = 754.15(2) Å^3, Z = 2, D_{calc} = 2.026 gcm^{-3}, F(000) = 444, μ = 18.34 mm^{-1}, T = 120.0(1) K, θ_{max} = 74.5°, 843 total reflections, 843 with $I_o > 2\sigma(I_o)$, R_{int} = 0.022, 843 data, 75 parameters, no restraints, GooF = 1.12, 0.45 < dΔρ < -0.58 eÅ^{-3}, $R[F^2 > 2\sigma(F^2)]$ = 0.018, $wR(F^2)$ = 0.045.

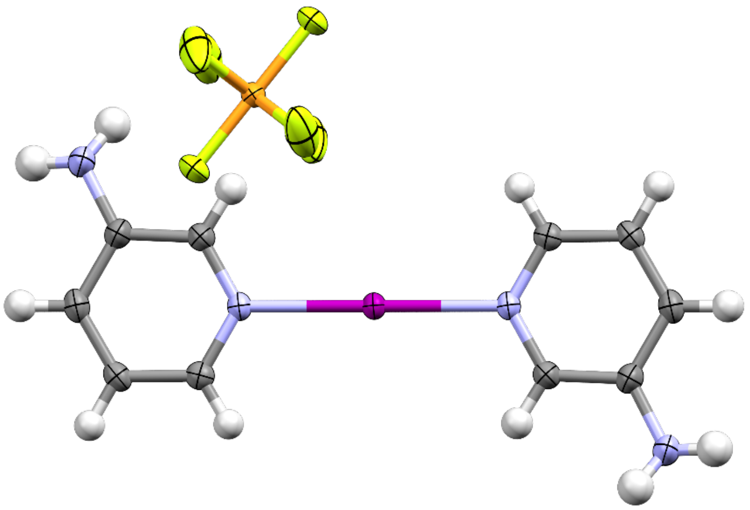

Figure S7: The crystal structure of **3b**. Colour key: purple = iodine, orange = phosphorus, lime green = fluorine, blue = nitrogen, dark grey = carbon, white = hydrogen.

[I(3-NMe$_2$py)$_2$]PF$_6$ (**4b**):

^{1}H NMR (500 MHz, CD$_3$CN) δ 8.05 (d, *J* = 2.5 Hz, 2H), 7.99 (d, *J* = 5.0 Hz, 2H), 7.41 (dd, *J* = 8.7, 2.1 Hz, 2H), 7.31 (dd, *J* = 8.8, 5.2 Hz, 2H), 3.01 (s, 12H); ^{15}N NMR (HMBC, CD$_3$CN) δ -174.0 (pyridinic), -330.0 (NMe$_2$).

^{1}H NMR (500 MHz, CD$_2$Cl$_2$) δ 7.97 (d, *J* = 2.4 Hz, 2H), 7.96 (d, *J* = 4.1 Hz, 2H), 7.32 – 7.25 (m, 4H), 3.05 (s, 12H); ^{15}N NMR (HMBC, CD$_2$Cl$_2$) δ -173.4 (pyridinic), -329.5 (NMe$_2$).

Crystals suitable for single crystal X-ray diffraction were obtained by evaporation of a DCM solution of **4b**. Crystal data for **4b**: CCDC-2253729, [C$_{14}$H$_{20}$IN$_4$]PF$_6$, M = 516.21, colourless block, 0.08 × 0.11 × 0.18 mm, orthorhombic, space group *Pban*, a = 16.1759(13) Å, b = 7.3677(6) Å, c = 15.7389(14) Å, V = 1875.7(3) Å^3, Z = 4, D_{calc} = 1.828 gcm^{-3}, F(000) = 1016, μ = 1.86 mm^{-1}, T = 120.0(1) K, θ_{max} = 26.4°, 1914 total reflections, 1408 with $I_o > 2\sigma(I_o)$, R_{int} = 0.039, 1914 data, 136 parameters, 30 restraints, GooF = 1.25, 2.68 < dΔρ < -0.83 eÅ^{-3}, $R[F^2 > 2\sigma(F^2)]$ = 0.077, $wR(F^2)$ = 0.183.

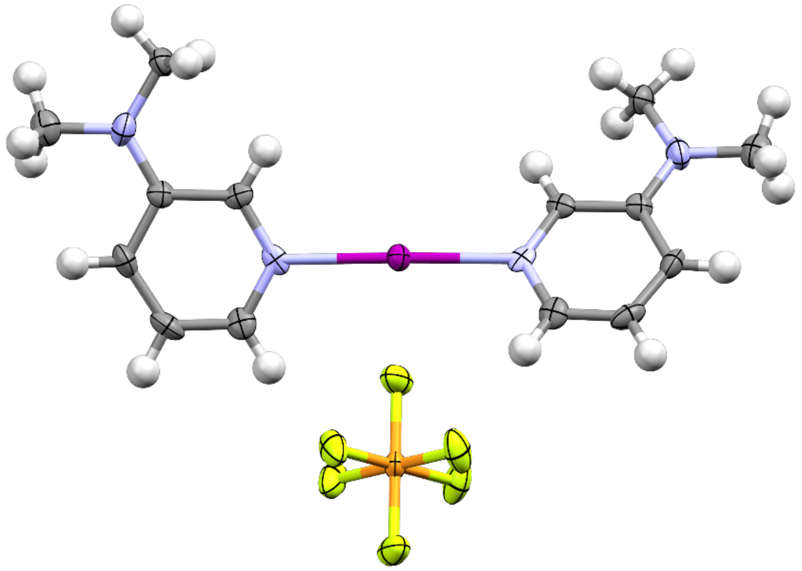

Figure S8: The crystal structure of **4b**. Colour key: purple = iodine, orange = phosphorus, lime green = fluorine, blue = nitrogen, dark grey = carbon, white = hydrogen.

The hydronium hexafluorophosphate complex of 3-NMe$_2$py (**4**) was also isolated in (manually separated) trace amounts from crystallisation attempts of **4b**.

[H(3-NMe$_2$py)$_2$]PF$_6$ (**4f**):

^{1}H NMR (500 MHz, CD$_3$CN) δ 7.99 – 7.82 (br.m, 4H), 7.66 – 7.53 (br.m, 4H), 3.04 (s, 12H); ^{15}N NMR (HMBC, CD$_3$CN) δ -161.2 (pyridinic), -327.3 (NMe$_2$).

A trace amount of crystals suitable for single crystal X-ray diffraction were obtained from a DCM:MeCN (7:1) solution of **4b** vapour diffused with TBME. Crystal data for **4f**: CCDC-2253730, [C$_{14}$H$_{21}$N$_4$]PF$_6$, M = 390.32, colourless plate, 0.02 × 0.13 × 0.18 mm, monoclinic, space group *C*2/*c*, a = 14.7998(7) Å, b = 16.5871(7) Å, c = 7.0685(4) Å, β = 91.101(5)°, V = 1734.90(15) Å^3, Z = 4, D_{calc} = 1.494 gcm^{-3}, F(000) = 808, μ = 2.04 mm^{-1}, T = 120.0(1) K, θ_{max} = 74.4°,

1786 total reflections, 1571 with $I_o > 2\sigma(I_o)$, R_{int} = 0.030, 1786 data, 126 parameters, 15 restraints, GooF = 1.12, 0.81 < dΔρ < -0.45 eÅ^{-3}, $R[F^2 > 2\sigma(F^2)]$ = 0.045, $wR(F^2)$ = 0.134.

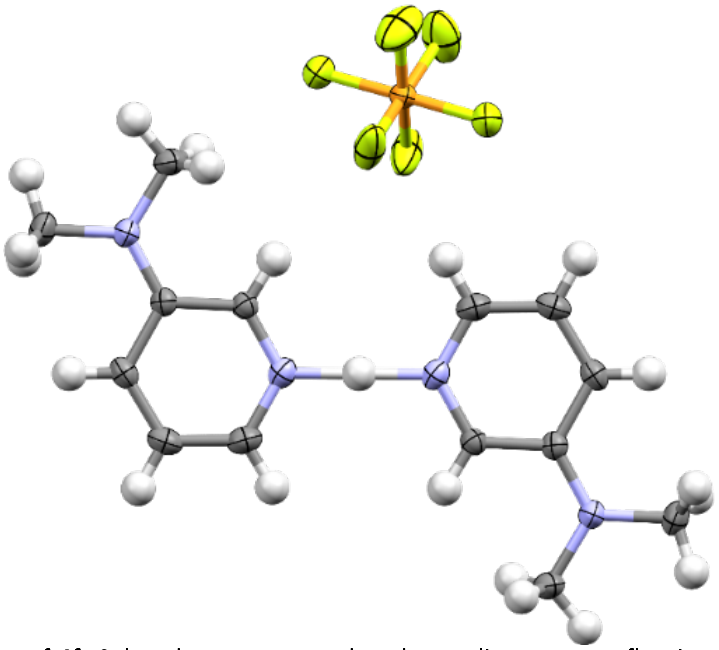

Figure S9: The crystal structure of **4f**. Colour key: orange = phosphorus, lime green = fluorine, blue = nitrogen, dark grey = carbon, white = hydrogen.

[I(3-CNpy)$_2$]PF$_6$ (**5b**):

The ^{1}H NMR spectra of freshly prepared samples of **5b** closely mirrored those of the free ligand **5**. It is presumed as a weak Lewis base that **5** is highly labile in solution, and therefore those spectroscopic values will not be reported herein. This was supported by the ^{1}H-^{15}N HMBC spectra, which in both the coordinating MeCN and non-coordinating CD_2Cl_2, were only observed to display extremely weak ^{15}N NMR shifts approximately matching those of the free ligand **5**.

Crystals suitable for single crystal X-ray diffraction were obtained by evaporation of a concentrated MeCN solution of **5b**. Crystal data for **5b**: CCDC-2253732, $[C_{12}H_8IN_4]PF_6$, M = 480.09, colourless plate, 0.01 × 0.08 × 0.18 mm, monoclinic, space group *C2/m*, a = 14.1185(5) Å, b = 6.6659(2) Å, c = 8.4958(2) Å, β = 107.583(3)°, V = 762.21(4) Å^3, Z = 2, D_{calc} = 2.092 gcm^{-3}, F(000) = 460, μ = 18.20 mm^{-1}, T = 120.0(1) K, θ_{max} = 74.4°, 857 total reflections, 855 with $I_o > 2\sigma(I_o)$, R_{int} = 0.050, 857 data, 90 parameters, no restraints, GooF = 1.18, 0.79 < dΔρ < -0.75 eÅ^{-3}, $R[F^2 > 2\sigma(F^2)]$ = 0.023, $wR(F^2)$ = 0.063.

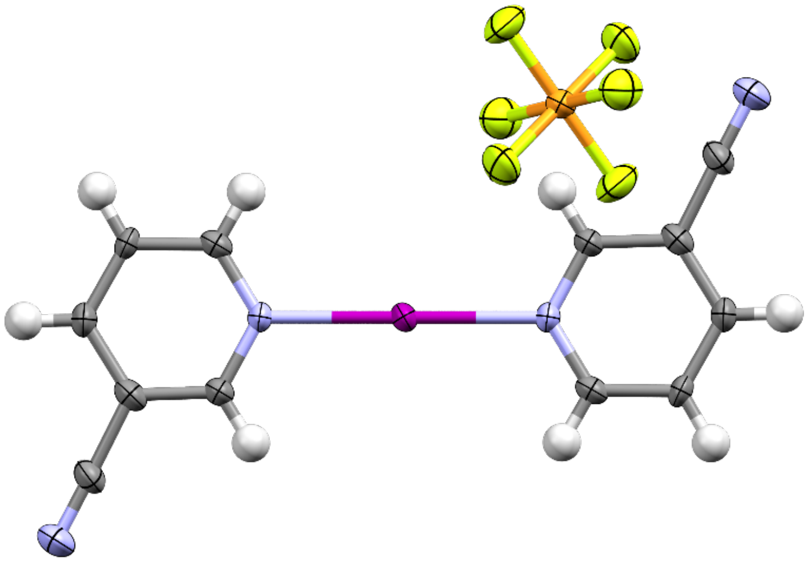

Figure S10: The crystal structure of **5b**. Colour key: purple = iodine, orange = phosphorus, lime green = fluorine, blue = nitrogen, dark grey = carbon, white = hydrogen.

Reaction Products of **1b**

[3-acetamido-1-(3-iodo-2-methylpentan-2-yl)pyridin-1-ium]PF_6 (1d):

^{1}H NMR (500 MHz, CD_2Cl_2) δ 9.31 (s, 1H), 8.96 (d, *J* = 8.5 Hz, 1H), 8.88 (br.s, 1H), 8.32 (d, *J* = 6.0 Hz, 1H), 7.93 (dd, *J* = 8.3, 6.5 Hz, 1H), 4.38 (dd, *J* = 9.5, 3.7 Hz, 1H), 2.28 (s, 3H), 2.03 (s, 3H), 1.97 (s, 3H), 1.81 – 1.71 (m, 2H), 1.17 (t, *J* = 7.0 Hz, 3H); ^{13}C NMR (126 MHz, CD_2Cl_2) δ 171.1, 141.1, 135.5, 134.2, 131.9, 128.2, 75.9, 49.6, 29.7, 26.1, 24.5, 24.4, 15.7; ^{15}N NMR (HMBC, CD_2Cl_2) δ -149.8 (pyridinic), -254.1 (amido).

Crystals suitable for single crystal X-ray diffraction were obtained from a DCM:MeCN (7:1) solution of **1b** vapour diffused with DIPE. Crystal data for **1d**: CCDC-2253721, $[C_{13}H_{20}IN_2O]PF_6$, M = 492.18, colourless block, 0.17 × 0.25 × 0.32 mm, triclinic, space group *P*-1 (No. 2), a = 7.3369(3) Å, b = 10.5036(4) Å, c = 12.8213(5) Å, α = 104.320(3)°, β = 92.689(3)°, γ = 108.984(3)°, V = 896.50(6) Å^3, Z = 2, D_{calc} = 1.823 gcm^{-3}, F(000) = 484, μ = 15.48 mm^{-1}, T = 120.0(1) K, θ_{max} = 74.5°, 3612 total reflections, 3567 with $I_o > 2\sigma(I_o)$, R_{int} = 0.026, 3612 data, 224 parameters, no restraints, GooF = 1.08, 1.02 < dΔρ < -1.02 eÅ^{-3}, $R[F^2 > 2\sigma(F^2)]$ = 0.029, $wR(F^2)$ = 0.077.

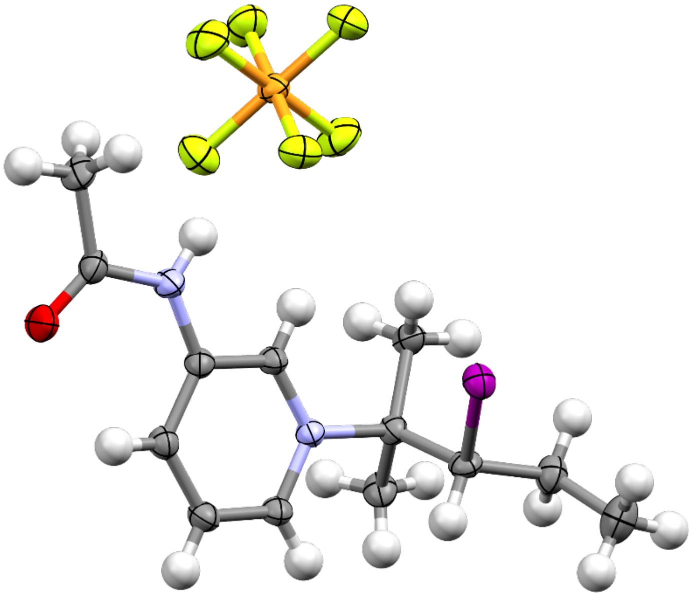

Figure S11: The crystal structure of **1d**. Colour key: purple = iodine, orange = phosphorus, lime green = fluorine, red = oxygen, blue = nitrogen, dark grey = carbon, white = hydrogen.

[H(3-AcNHpy)]PF_6 (1e):

^{1}H NMR (500 MHz, CD_3CN) δ 9.32 (s, 1H), 9.10 (br.s, 1H), 8.40 – 8.30 (m, 2H), 7.93 (dd, *J* = 8.4, 5.8 Hz, 1H), 2.17 (s, 3H); ^{15}N NMR (HMBC, CD_3CN) δ -180.1 (pyridinic), -253.1 (amido).

Crystals suitable for single crystal X-ray diffraction were obtained from a MeOH solution of **1b** vapour diffused with DIPE. Crystal data for **1e·1**: CCDC-2253722, $[C_7H_9N_2O]PF_6 \cdot C_7H_8N_2O$, M = 418.28, colourless block, 0.03 × 0.03 × 0.05 mm, triclinic, space group *P*-1 (No. 2), a = 7.2295(7) Å, b = 7.3178(7) Å, c = 9.2983(8) Å, α = 78.886(8)°, β = 68.794(9)°, γ = 66.718(9)°, V = 420.55(8) Å^3, Z = 1, D_{calc} = 1.652 gcm^{-3}, F(000) = 214, μ = 2.24 mm^{-1}, T = 120.0(1) K, θ_{max} = 74.4°,

1688 total reflections, 1482 with $I_o > 2\sigma(I_o)$, $R_{int} = 0.043$, 1688 data, 131 parameters, no restraints, GooF = 1.10, 0.37 < dΔρ < -0.62 eÅ^{-3}, $R[F^2 > 2\sigma(F^2)] = 0.048$, $wR(F^2) = 0.132$.

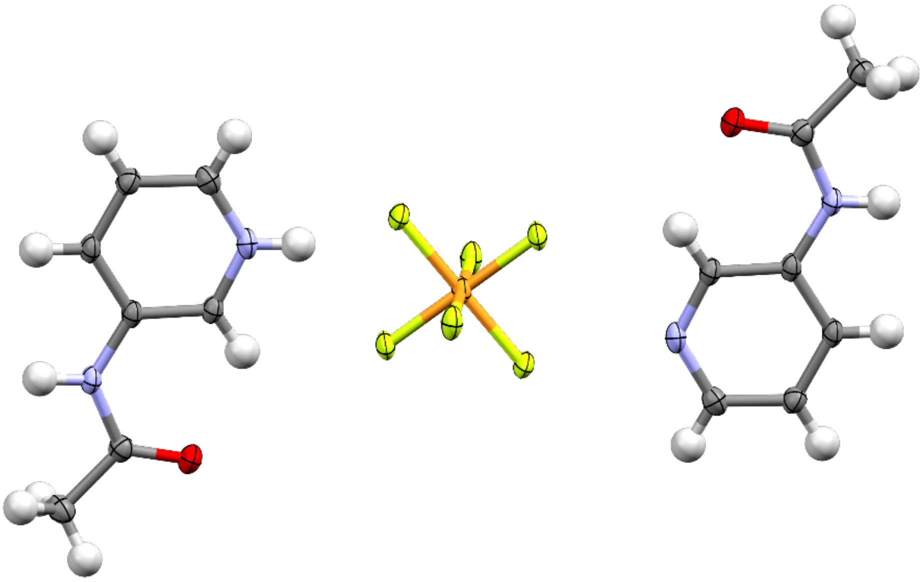

Figure S12: The crystal structure of **1e·1**. Colour key: orange = phosphorus, lime green = fluorine, red = oxygen, blue = nitrogen, dark grey = carbon, white = hydrogen.

Comparison Tables of ^{15}N NMR Chemical Shifts

Table S1: Comparison of the ^{15}N NMR chemical shifts in CD_3CN or CD_2Cl_2 (when possible) of the pyridinic nitrogen atoms of the 3-substituted pyridine ligands, and their silver(I) and iodine(I) complexes (in ppm).

**Compound/ Complex**	**Pyridinic ^{15}N NMR Chemical Shift(s) (δ_N)[§]**		
	**Ligands**	**Silver(I) Complexes (a)**	**Iodine(I) Complexes (b)**
3-AcNHpy (**1**)[1]	-63.7	-85.8	-174.5
3-Acpy (**2**)	-65.3 [-66.4[‡]]	-83.6 [Poor solubility[‡]]	-175.9 [-175.2[‡]]
3-NH_2py (**3**)	-64.0 [Poor solubility[‡]]	-98.8 [Poor solubility[‡]]	-173.9 [Poor solubility[‡]]
3-NMe_2py (**4**)	-65.3 [-67.4[‡]]	-103.3 [-132.3[‡]]	-174.0 [-173.4[‡]]
3-CNpy (**5**)	-63.2 [-63.5[‡]]	-68.8 [Poor solubility[‡]]	Not observed [Not observed[‡]]

[§] The accuracy of reported ^{15}N NMR chemical shifts is ±0.6 or ±0.8 ppm. [‡] Recorded in CD_2Cl_2.

Table S2: Comparison of the ^{15}N NMR chemical shifts of the pyridinic nitrogen atoms of the reaction products and side products reported herein (in ppm).

**Complex**	**Pyridinic ^{15}N NMR Chemical Shift(s) (δ_N)[§]**
**1c**[1]	[-154.6[‡]]
**1d**	[-149.8[‡]]
**1e**	-180.1
**4f**	-161.2

[§] The accuracy of reported ^{15}N NMR chemical shifts is ±0.6 or ±0.8 ppm. [‡] Recorded in CD_2Cl_2.

NMR Spectra

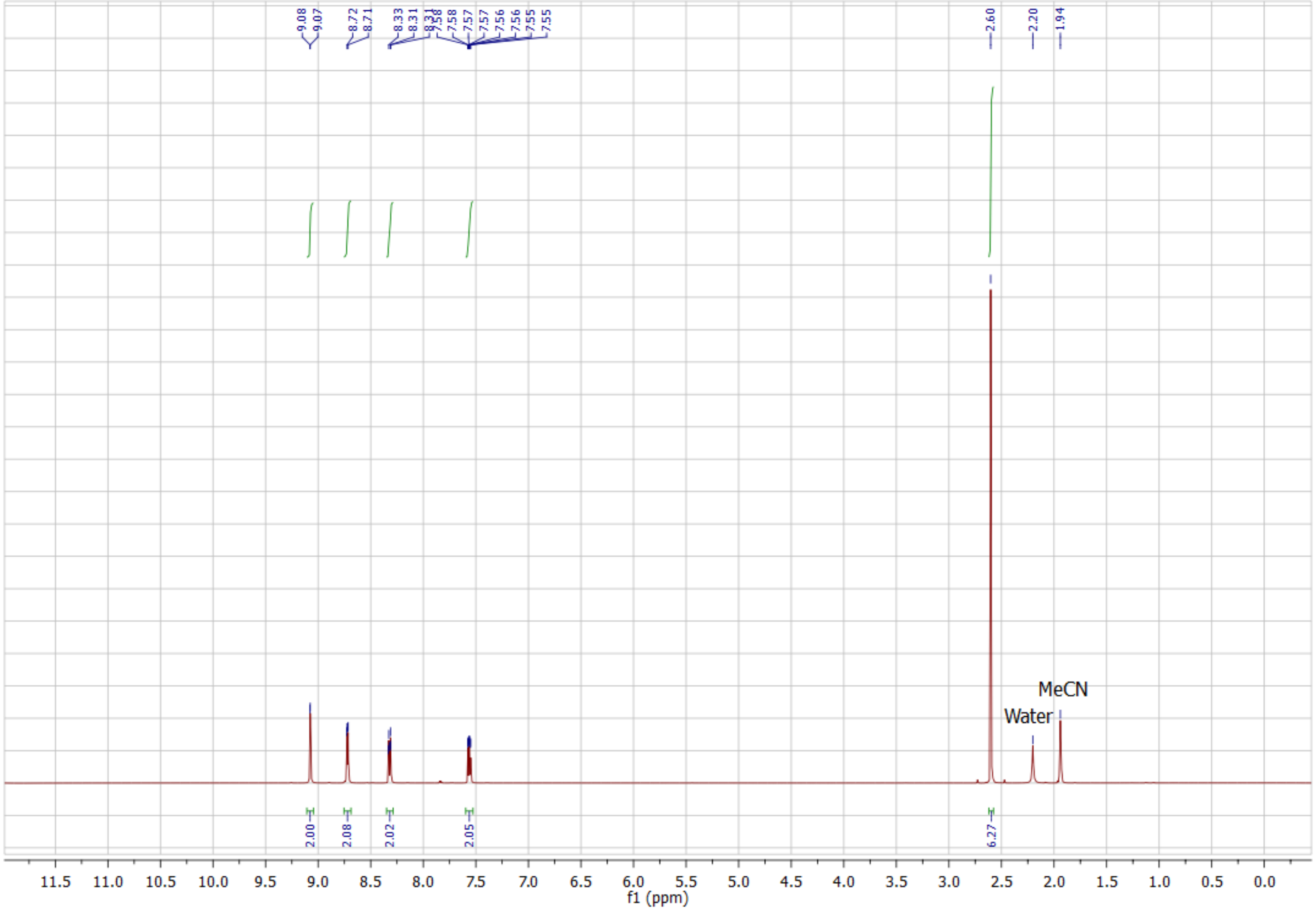

Figure S13: The ^{1}H NMR spectrum of complex **2a** in CD_3CN.

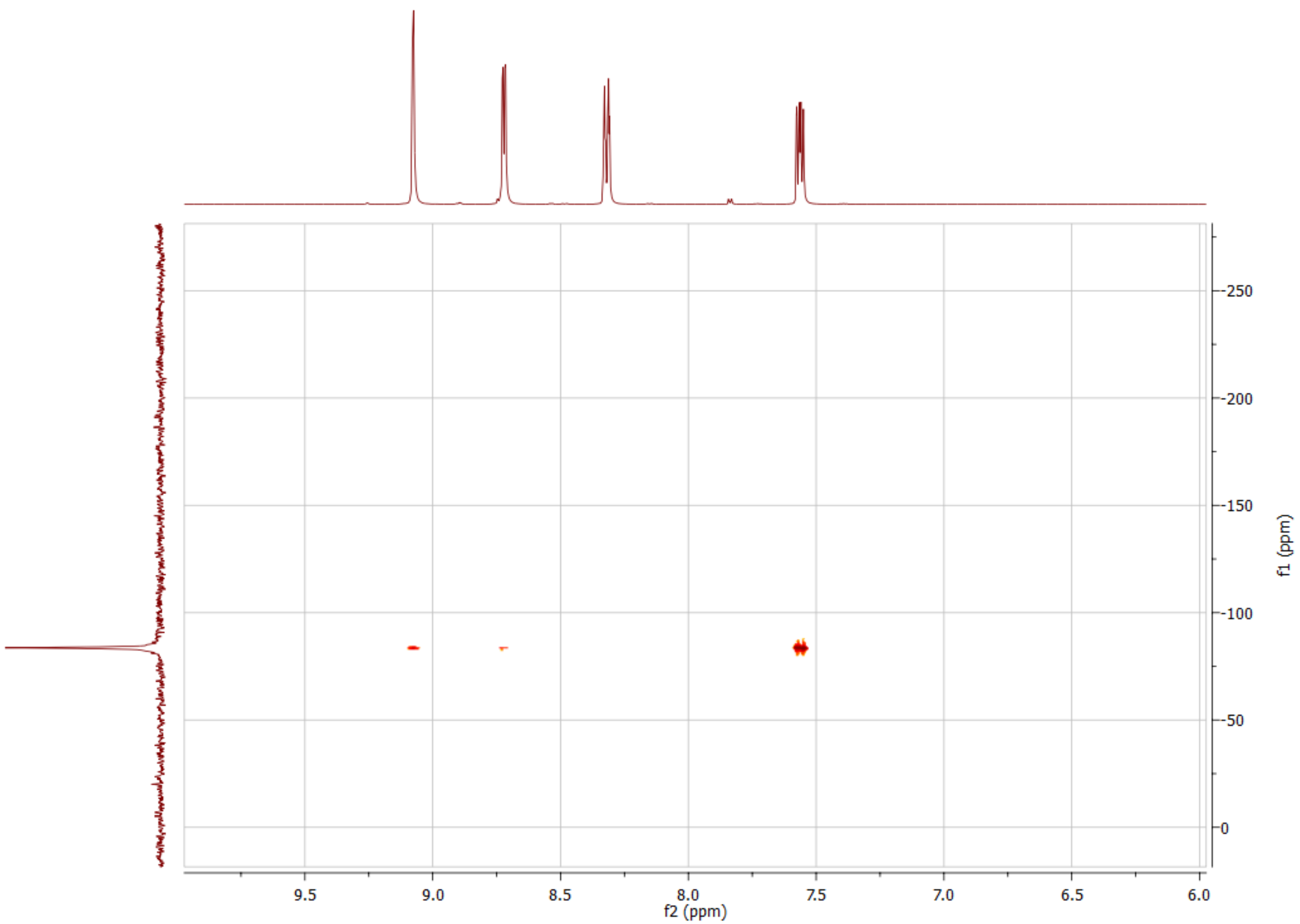

Figure S14: The ^{1}H-^{15}N HMBC spectrum of complex **2a** in CD_3CN.

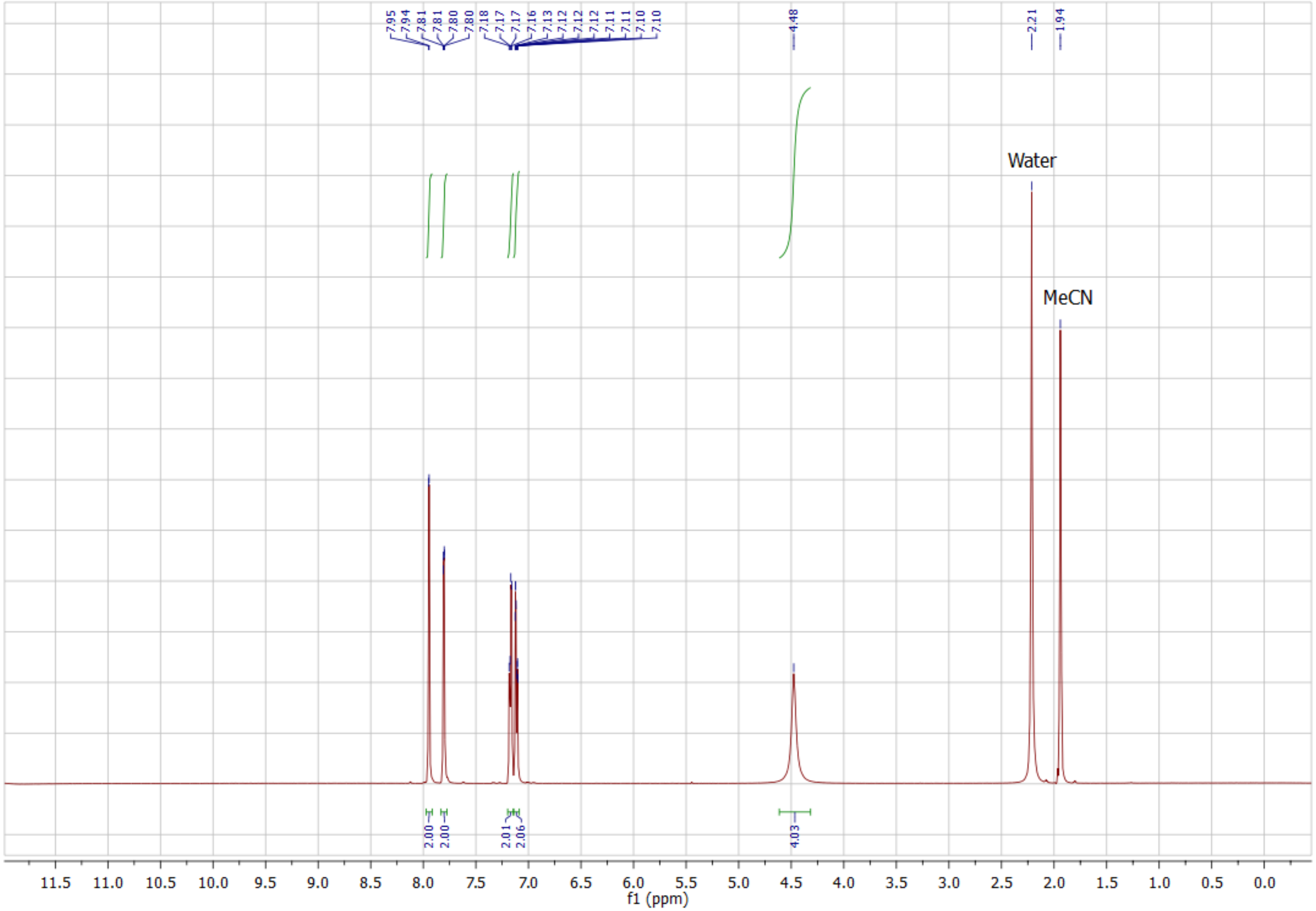

Figure S15: The ^{1}H NMR spectrum of complex **3a** in CD_3CN.

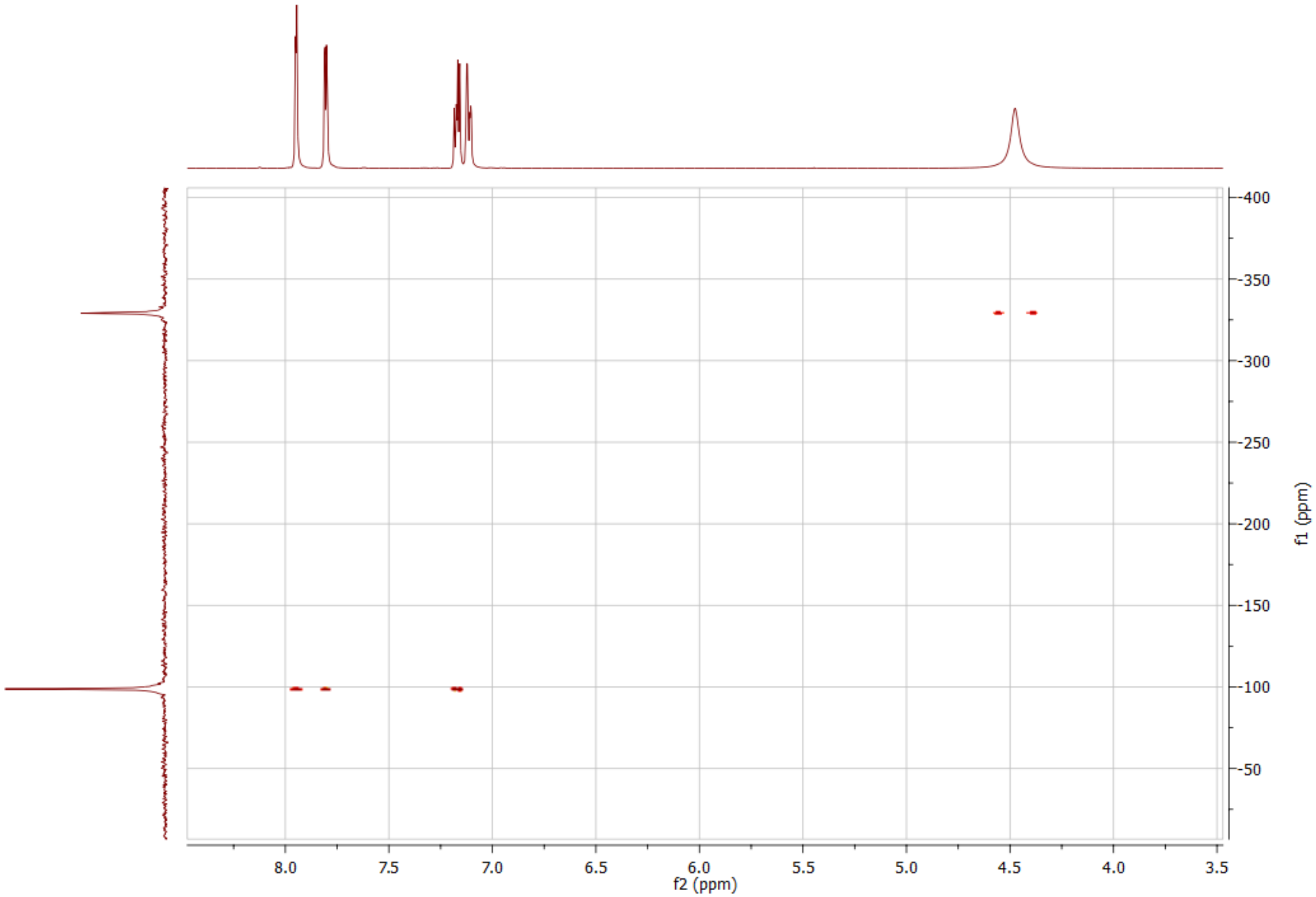

Figure S16: The ^{1}H-^{15}N HMBC spectrum of complex **3a** in CD_3CN.

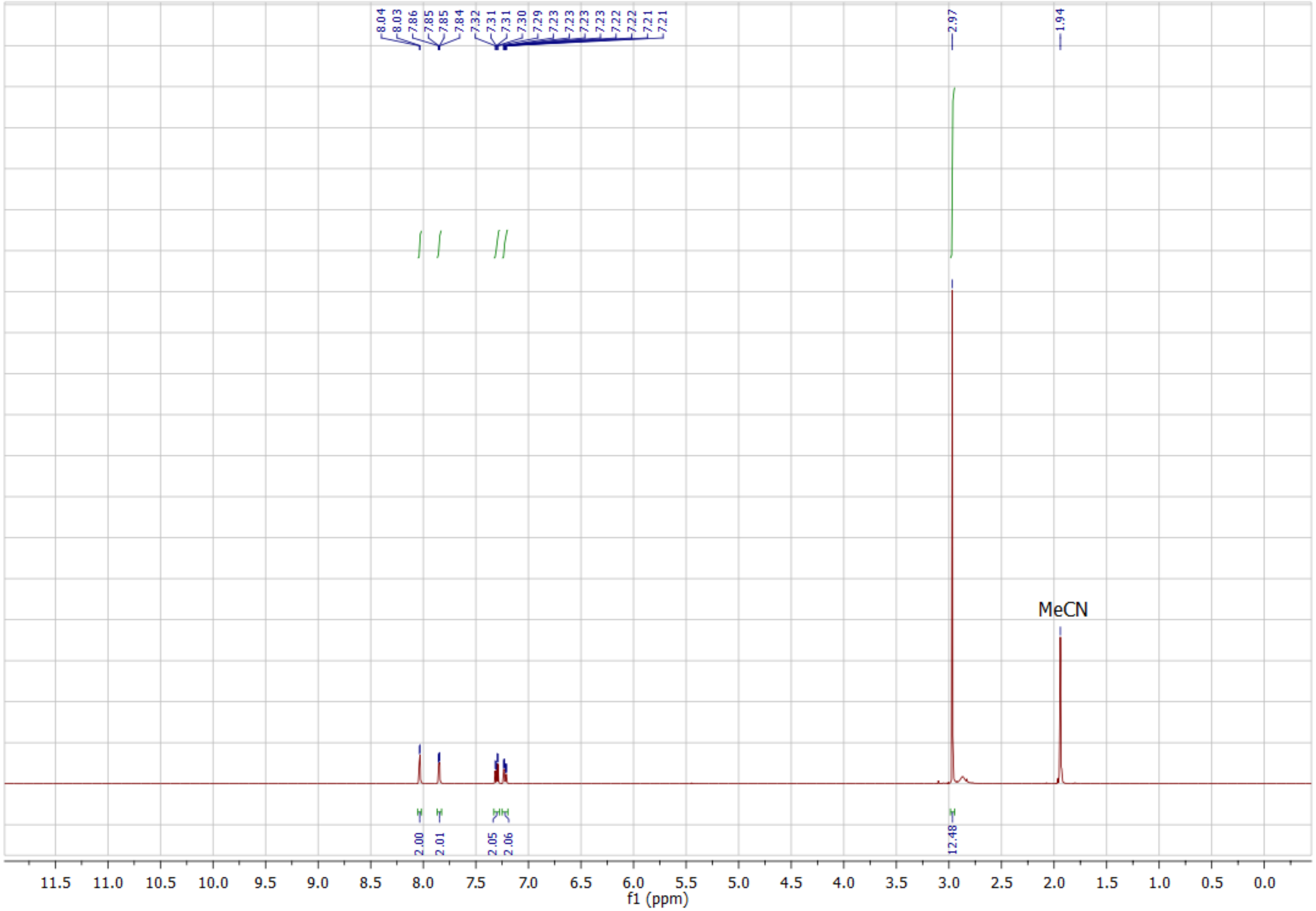

Figure S17: The ^{1}H NMR spectrum of complex **4a** in CD_3CN.

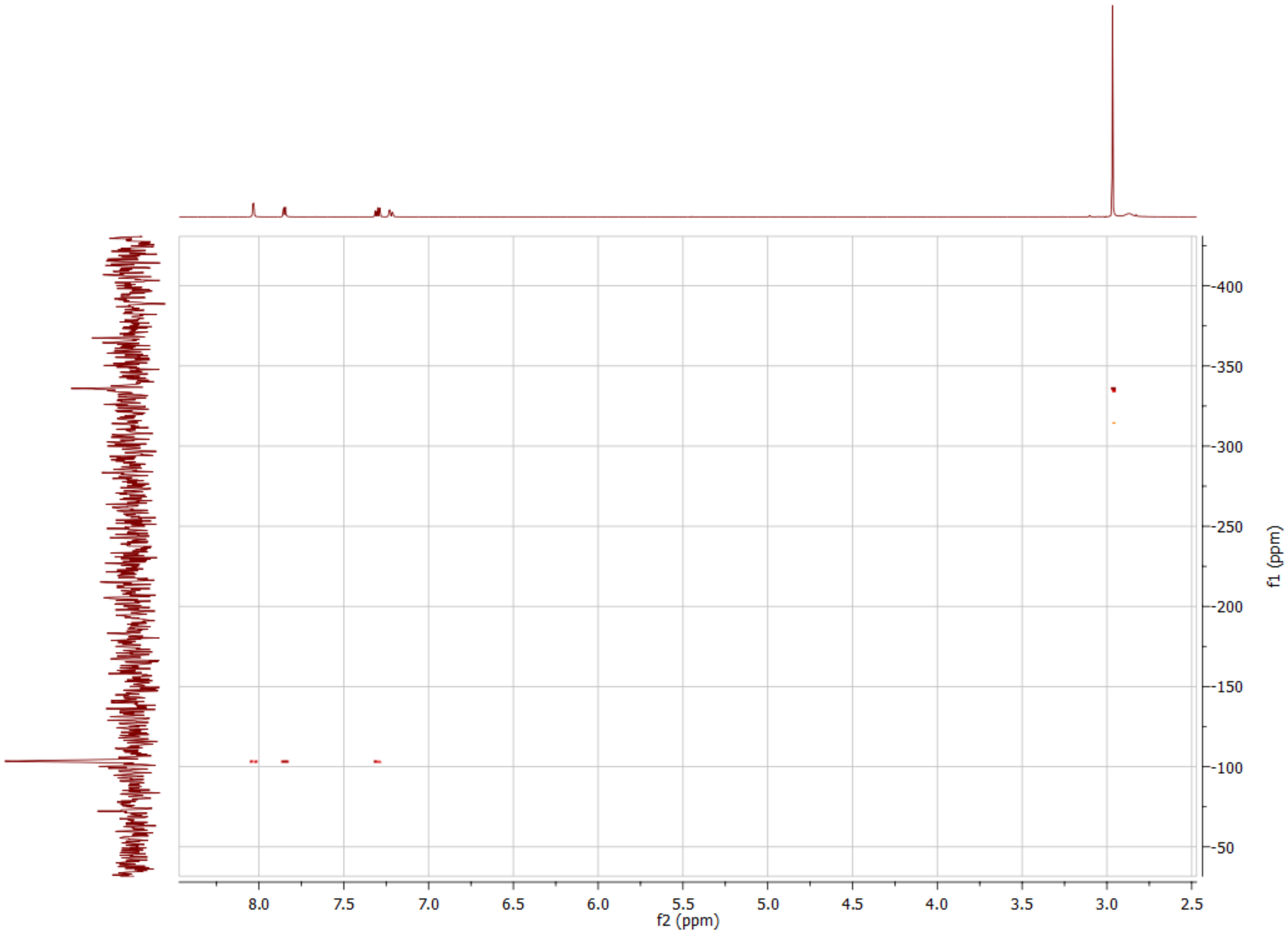

Figure S18: The ^{1}H-^{15}N HMBC spectrum of complex **4a** in CD_3CN.

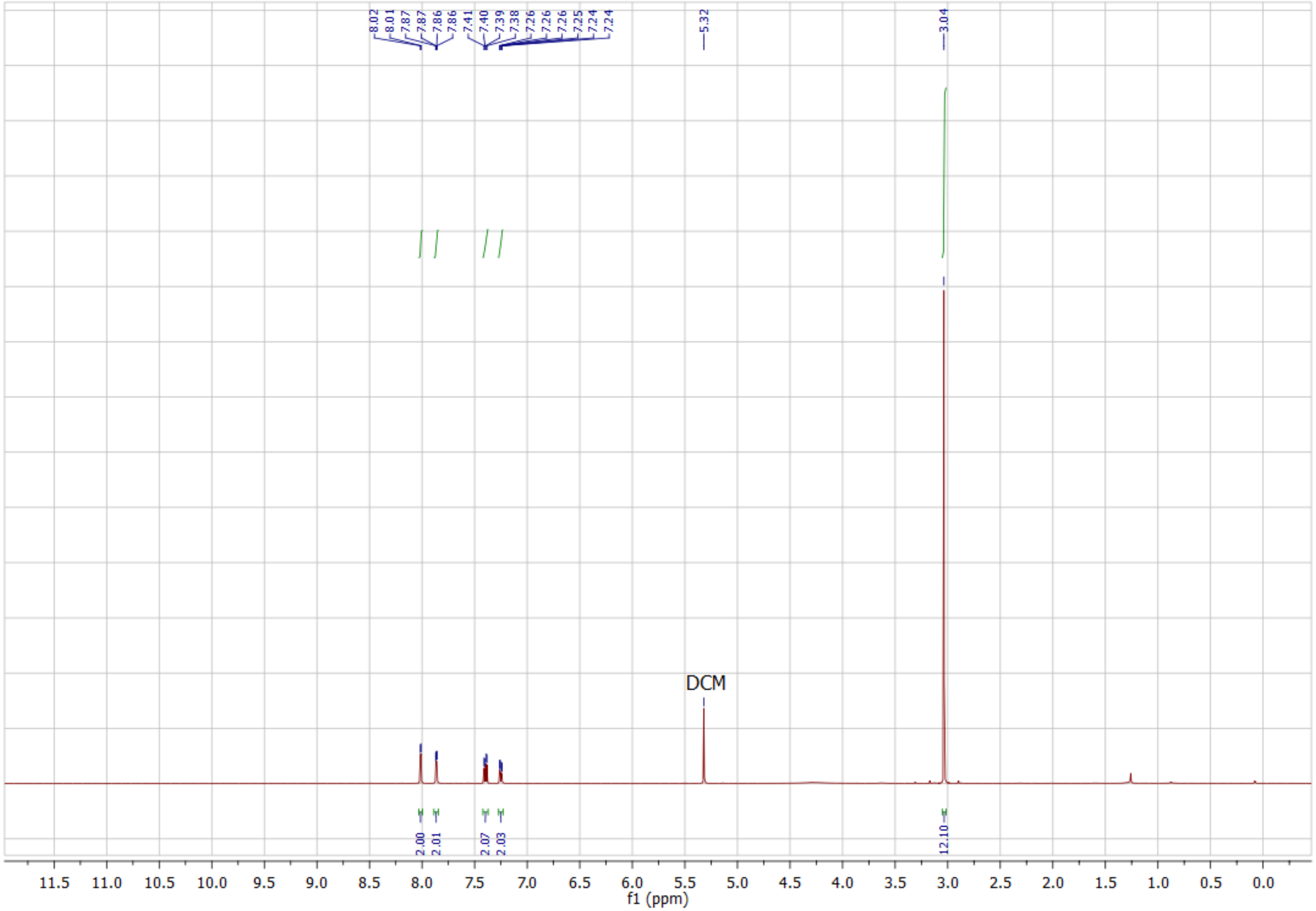

Figure S19: The ^{1}H NMR spectrum of complex **4a** in CD_2Cl_2.

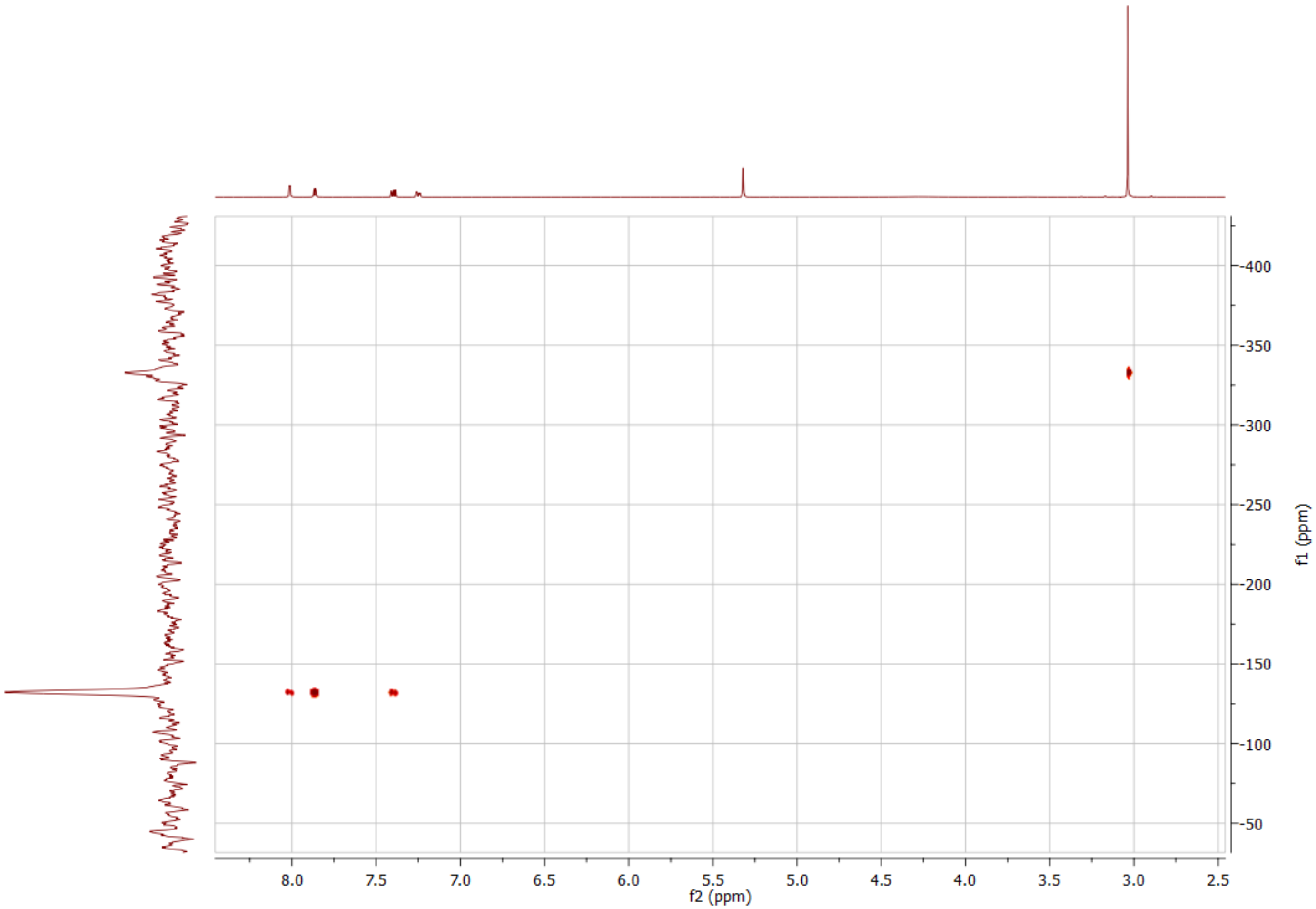

Figure S20: The ^{1}H-^{15}N HMBC spectrum of complex **4a** in CD_2Cl_2.

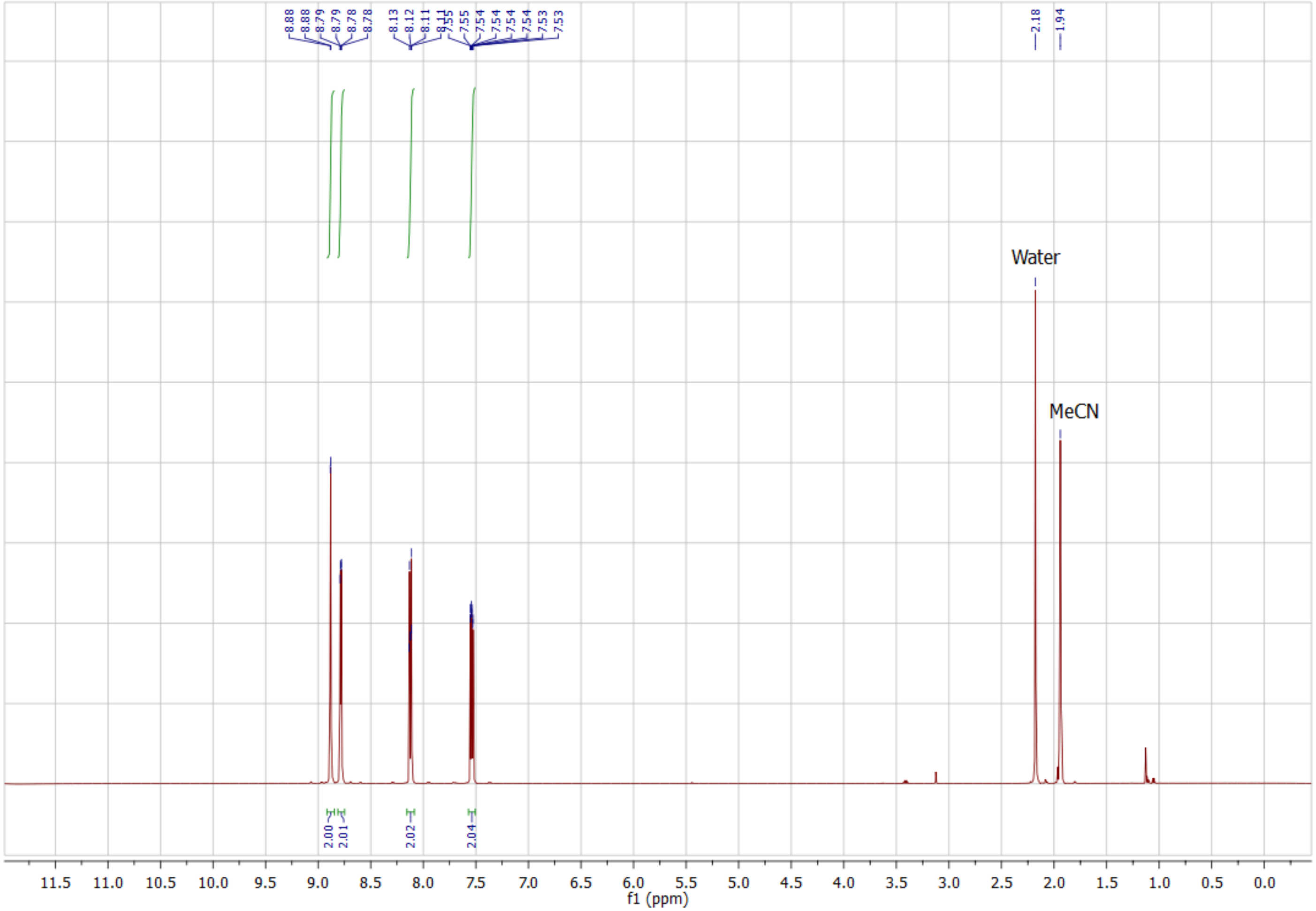

Figure S21: The ^{1}H NMR spectrum of complex **5a** in CD_3CN.

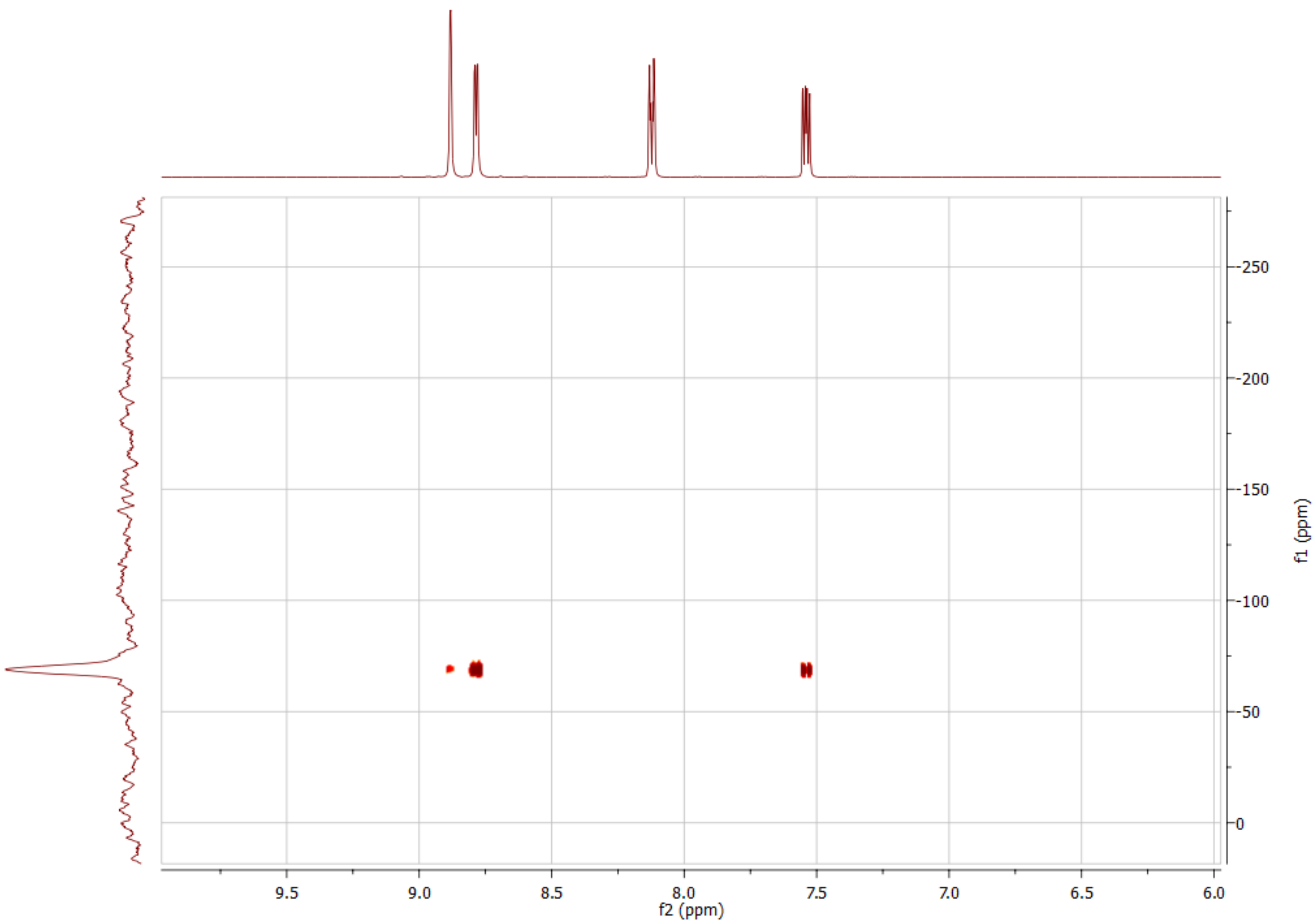

Figure S22: The ^{1}H-^{15}N HMBC spectrum of complex **5a** in CD_3CN.

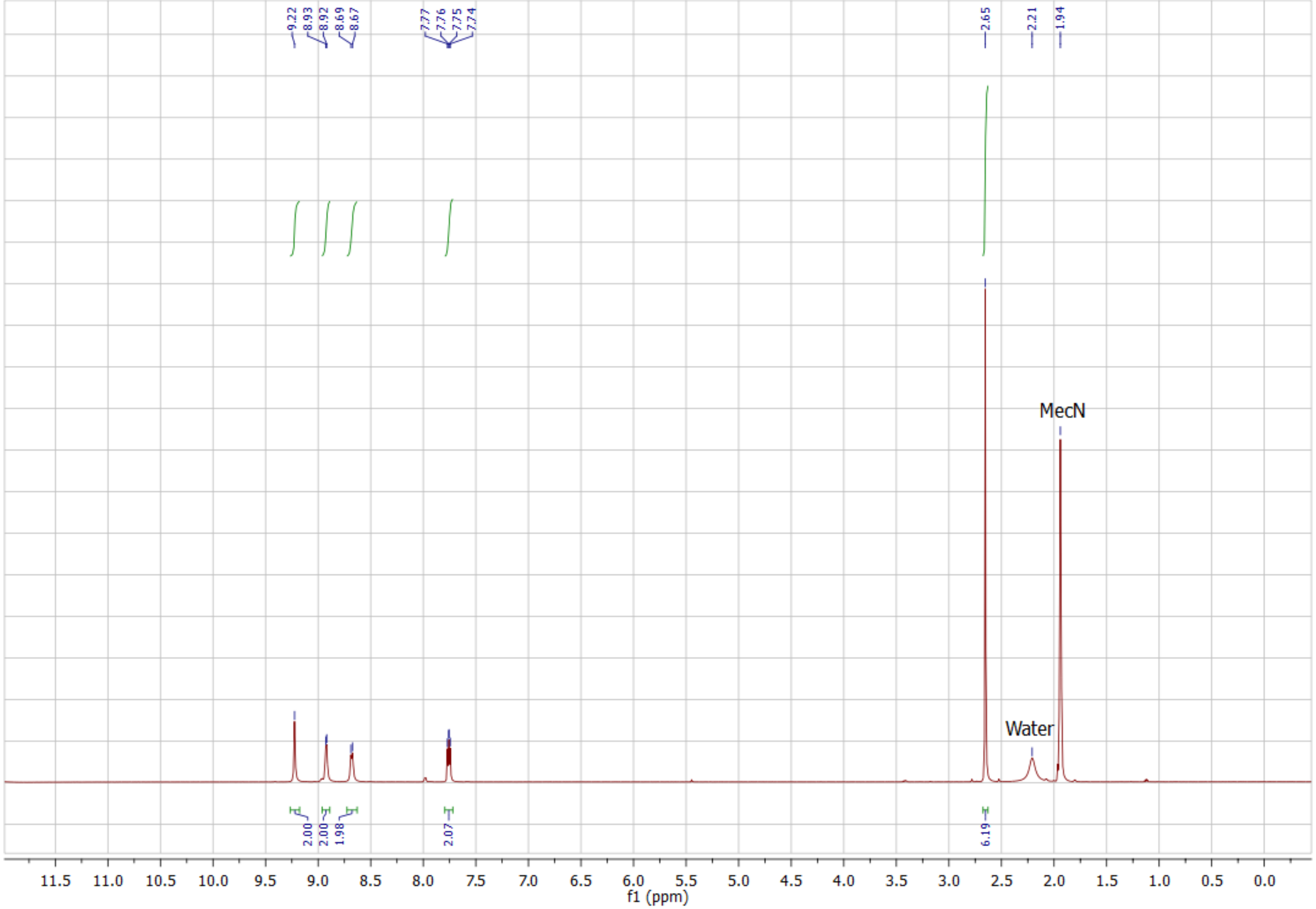

Figure S23: The ^{1}H NMR spectrum of complex **2b** in CD_3CN.

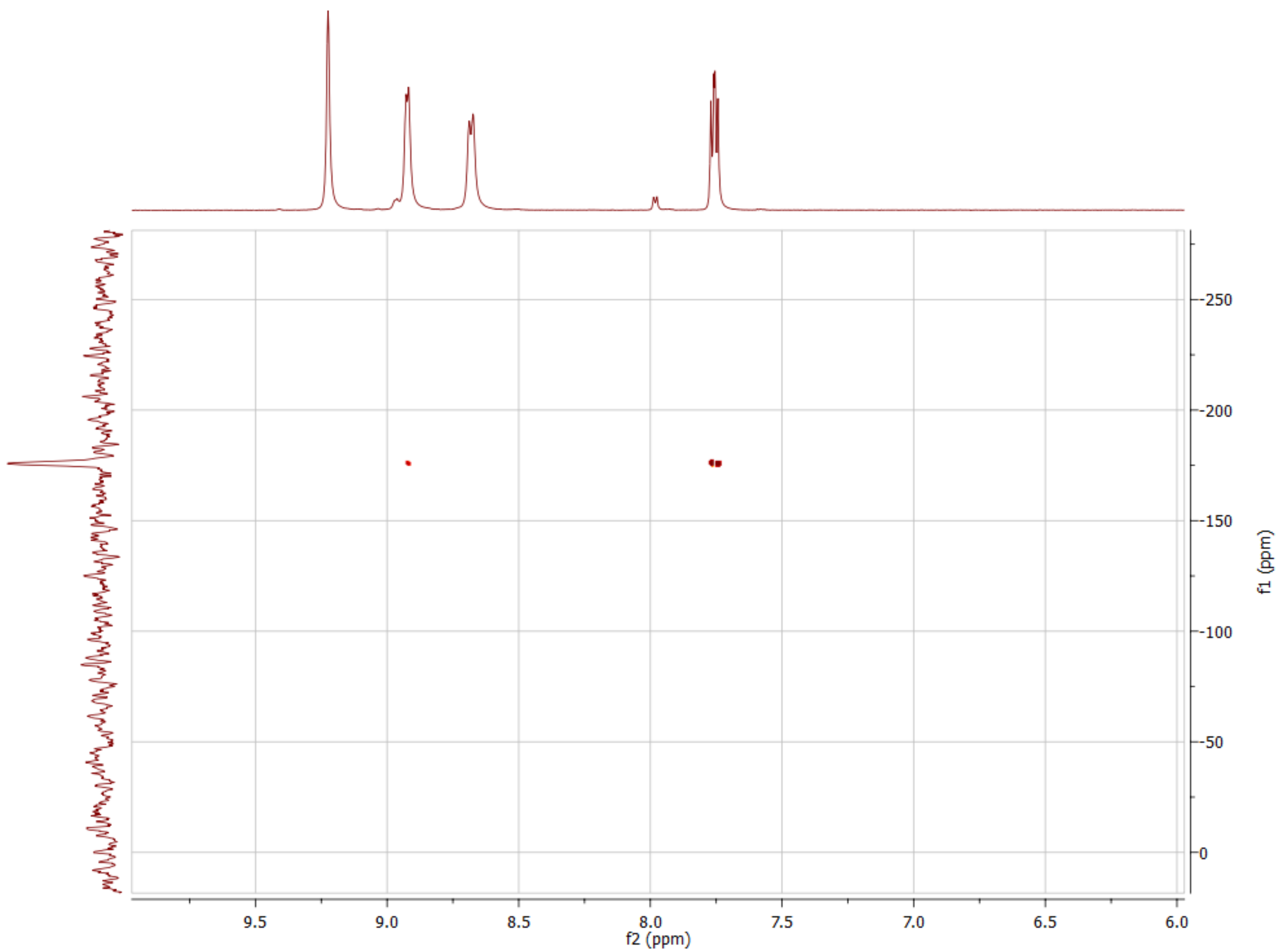

Figure S24: The ^{1}H-^{15}N HMBC spectrum of complex **2b** in CD_3CN.

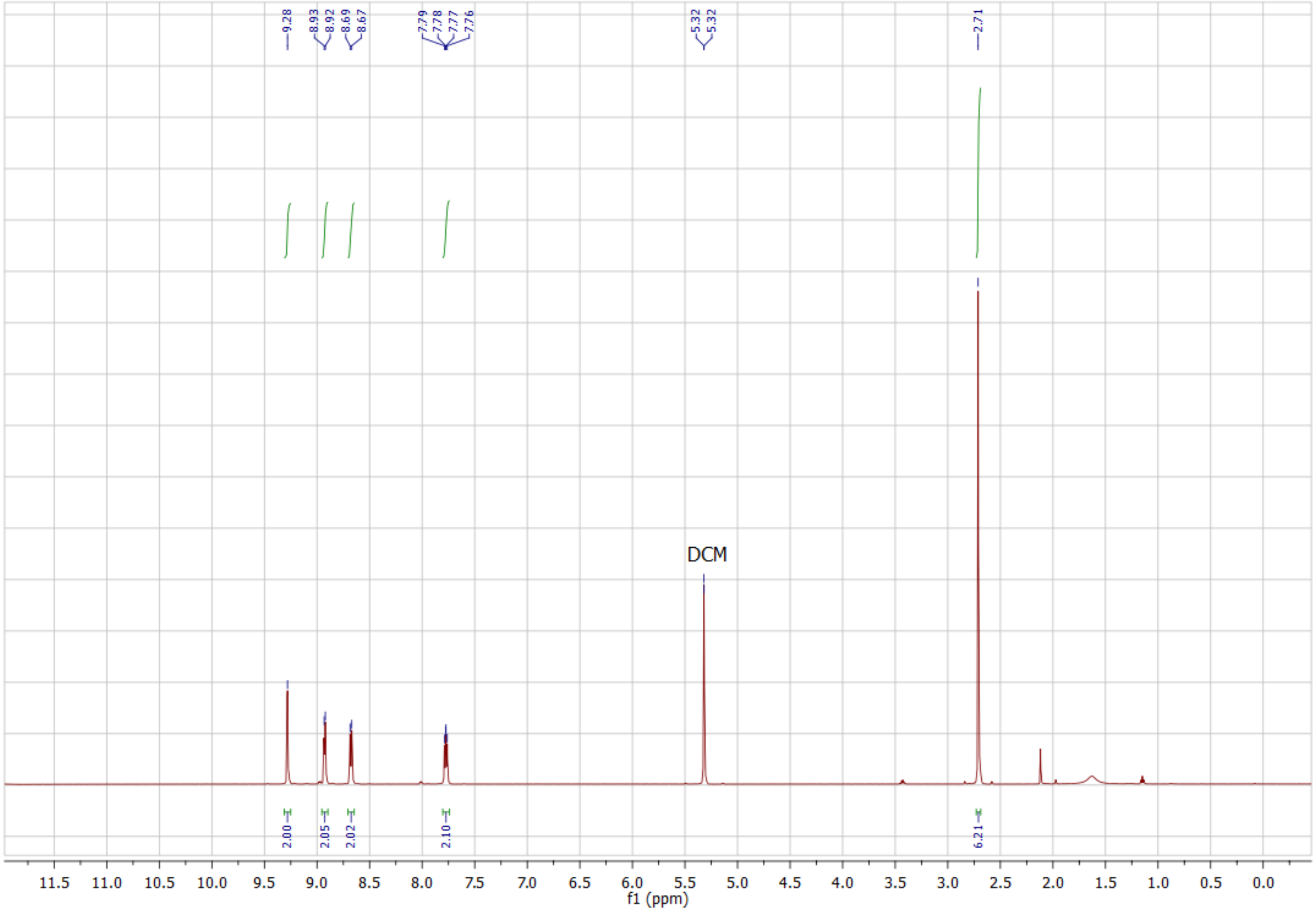

Figure S25: The ^{1}H NMR spectrum of complex **2b** in CD_2Cl_2.

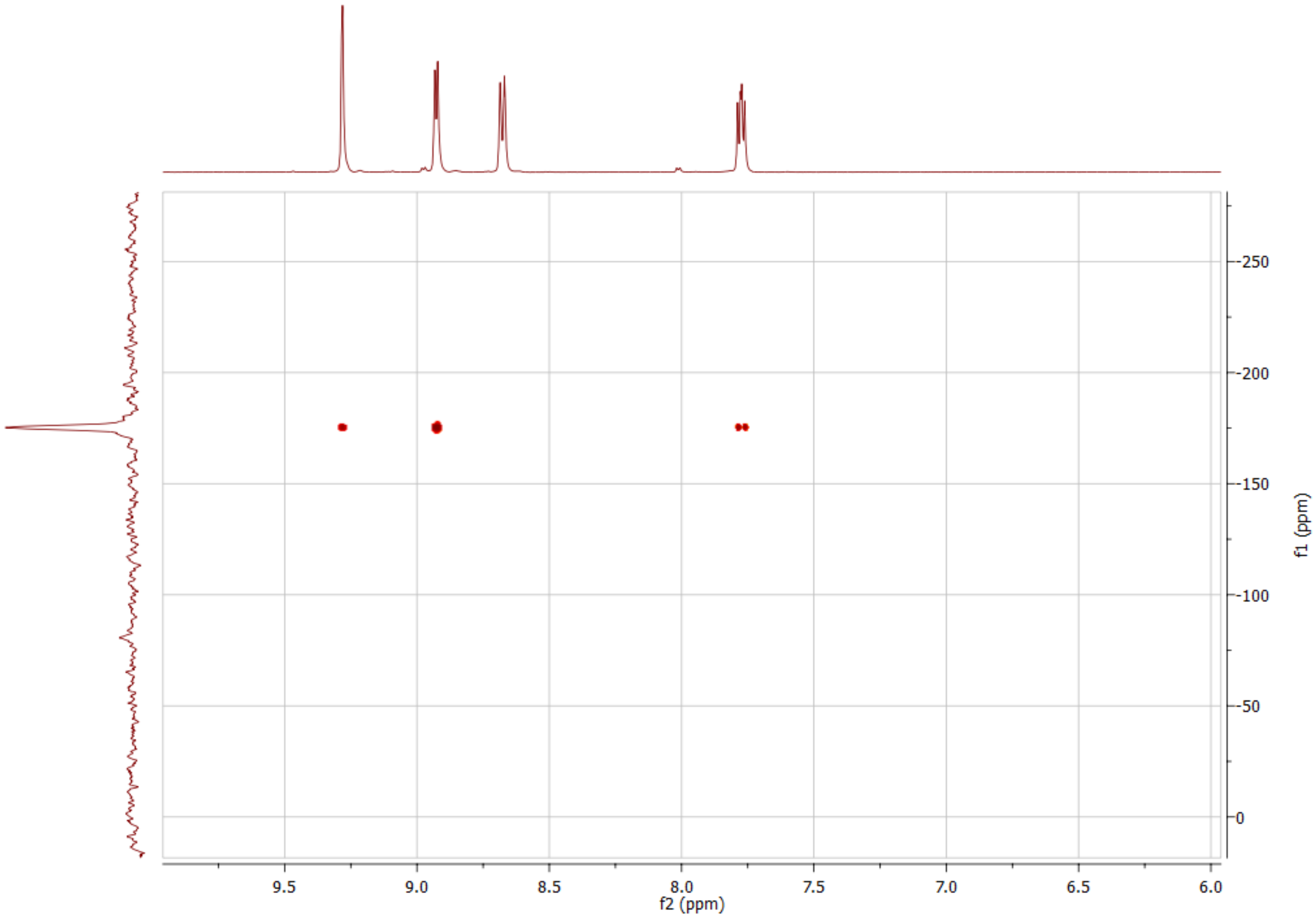

Figure S26: The ^{1}H-^{15}N HMBC spectrum of complex **2b** in CD_2Cl_2.

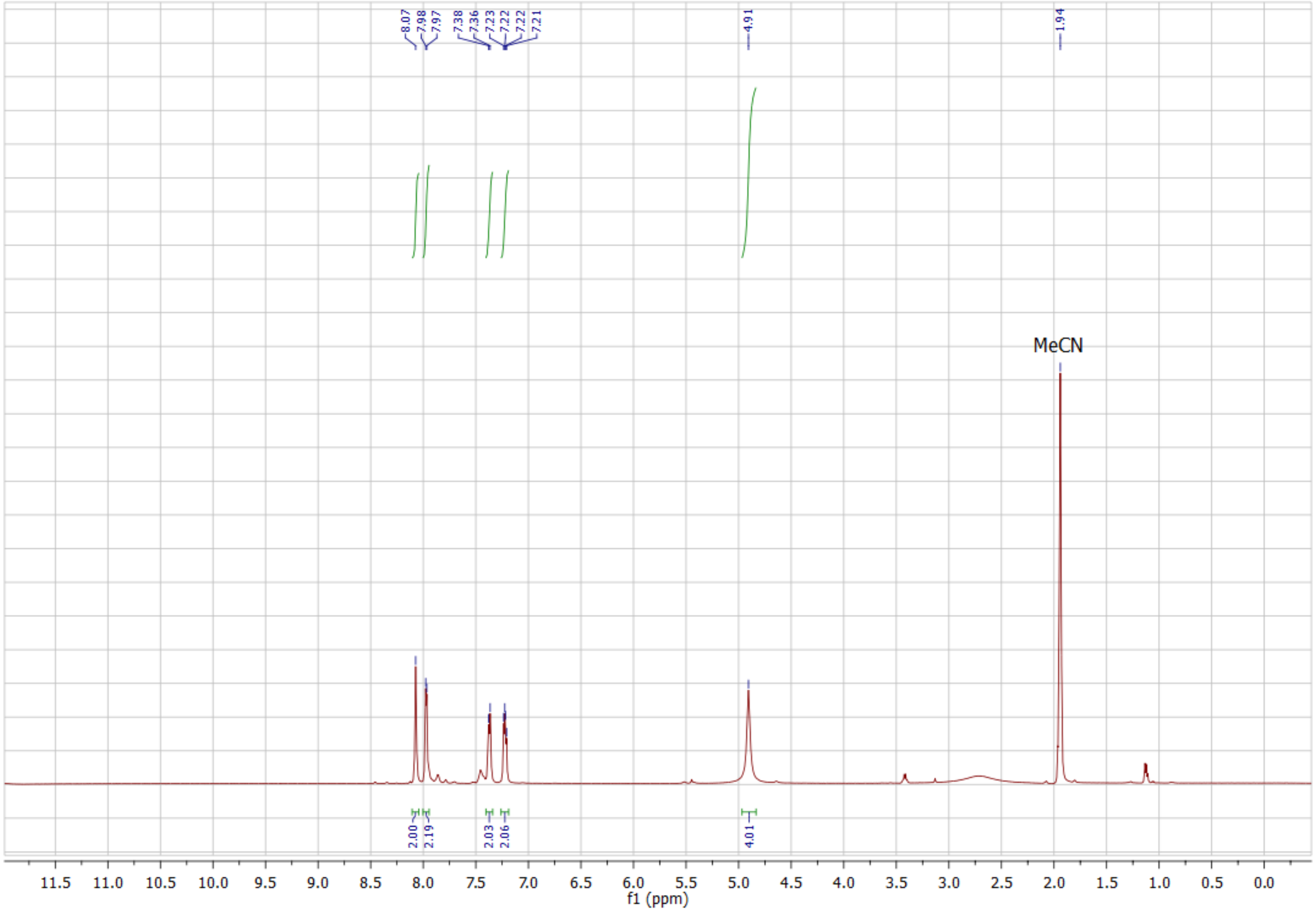

Figure S27: The ^{1}H NMR spectrum of complex **3b** in CD_3CN.

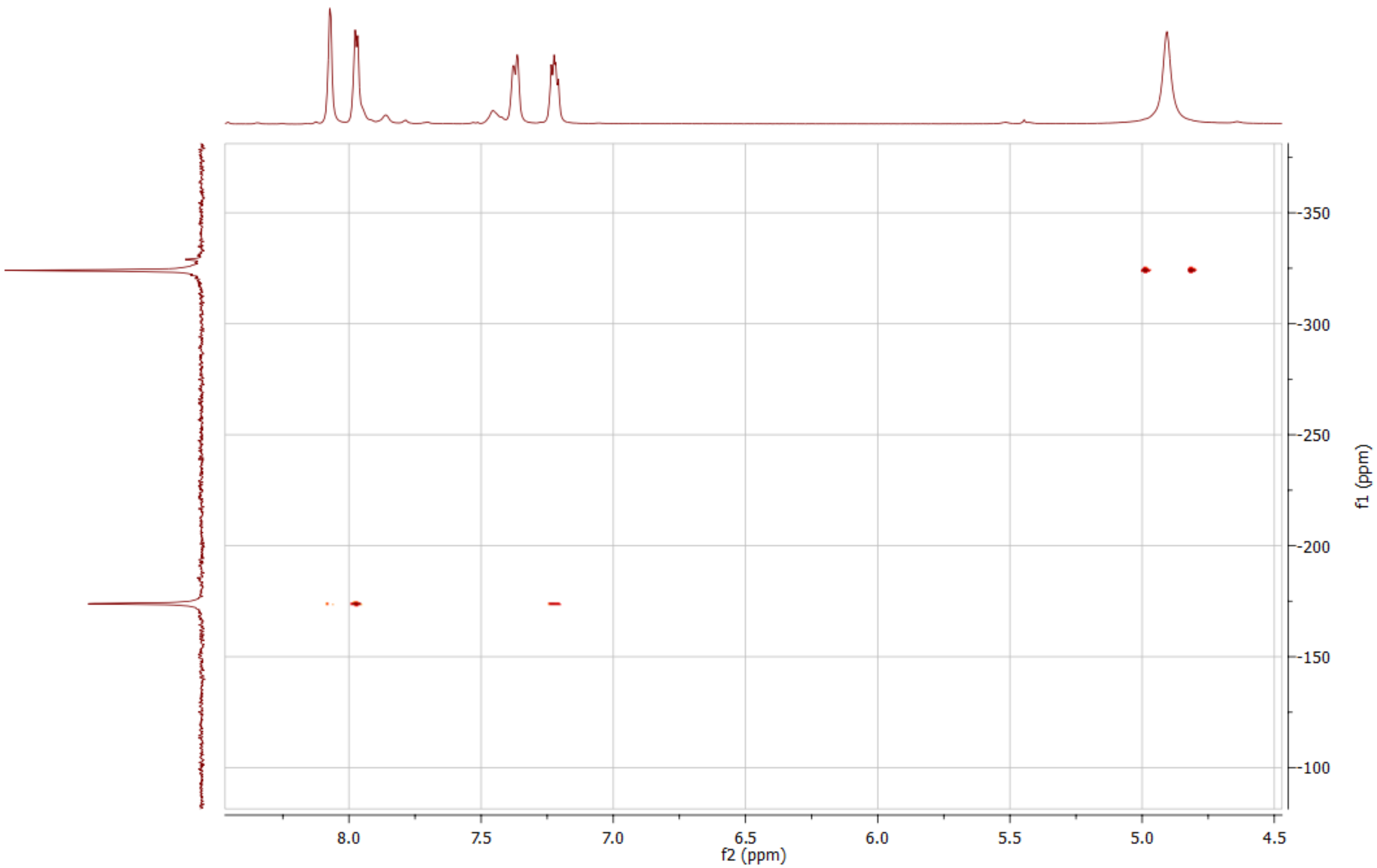

Figure S28: The ^{1}H-^{15}N HMBC spectrum of complex **3b** in CD_3CN.

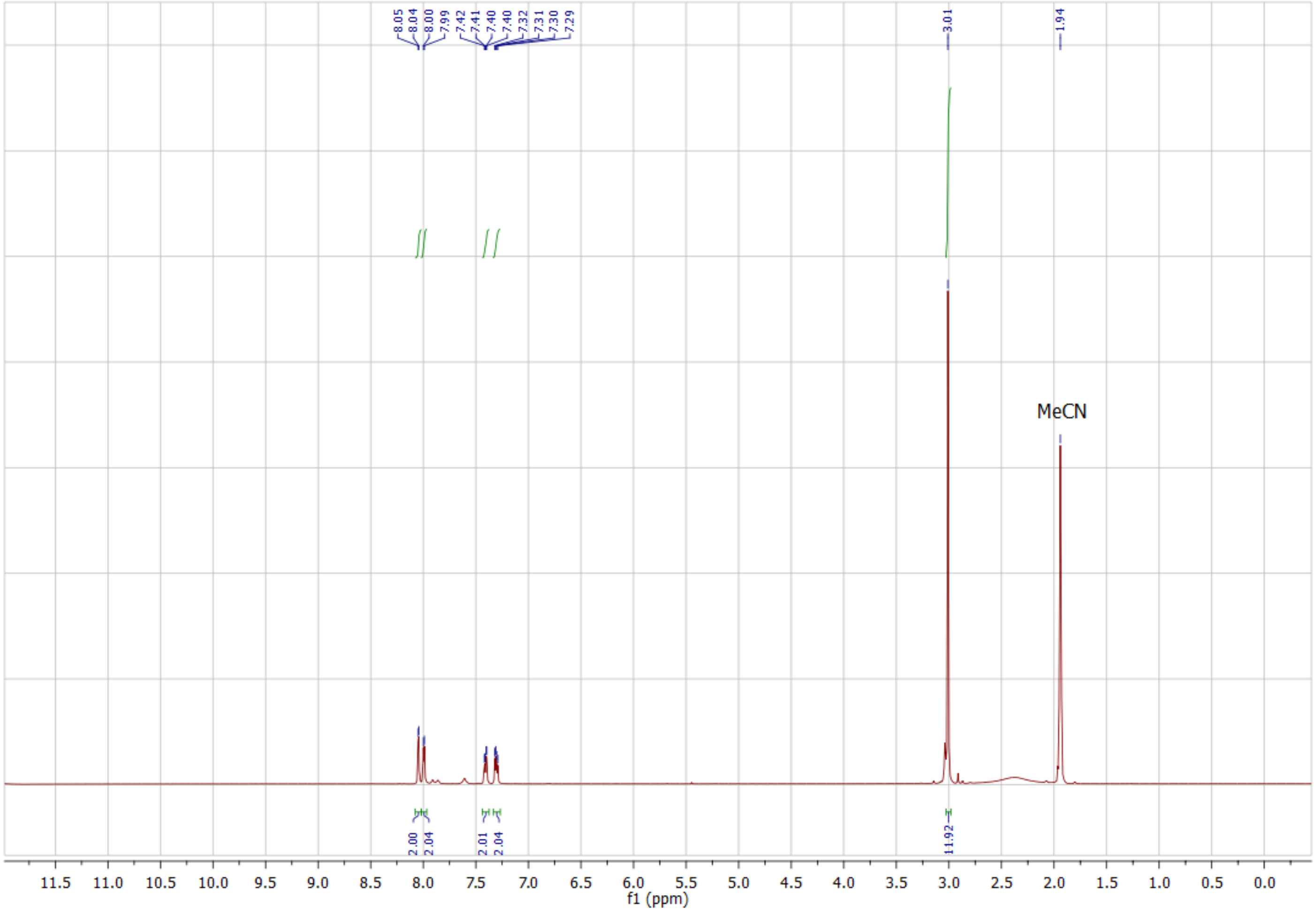

Figure S29: The ^{1}H NMR spectrum of complex **4b** in CD_3CN.

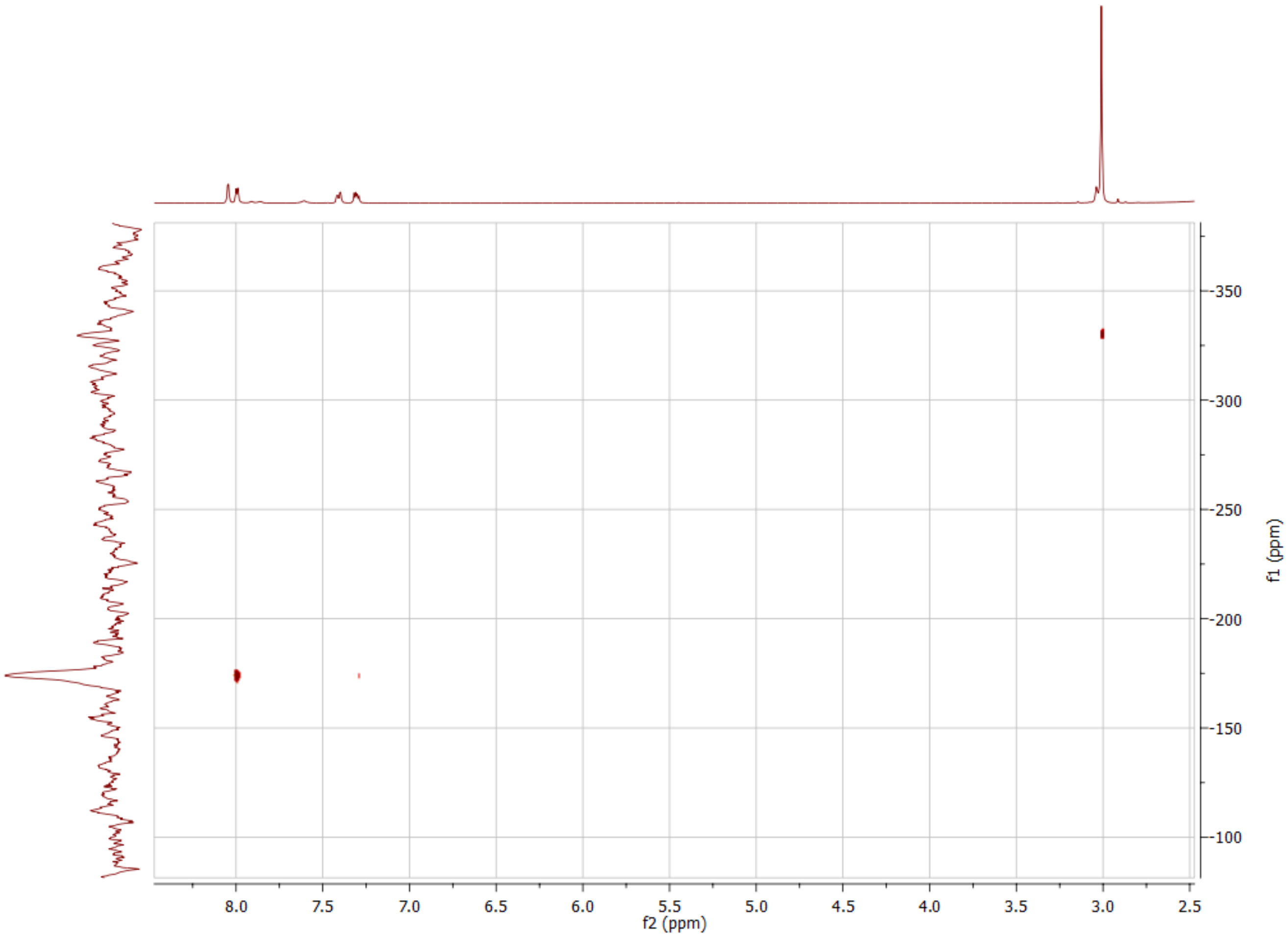

Figure S30: The ^{1}H-^{15}N HMBC spectrum of complex **4b** in CD_3CN.

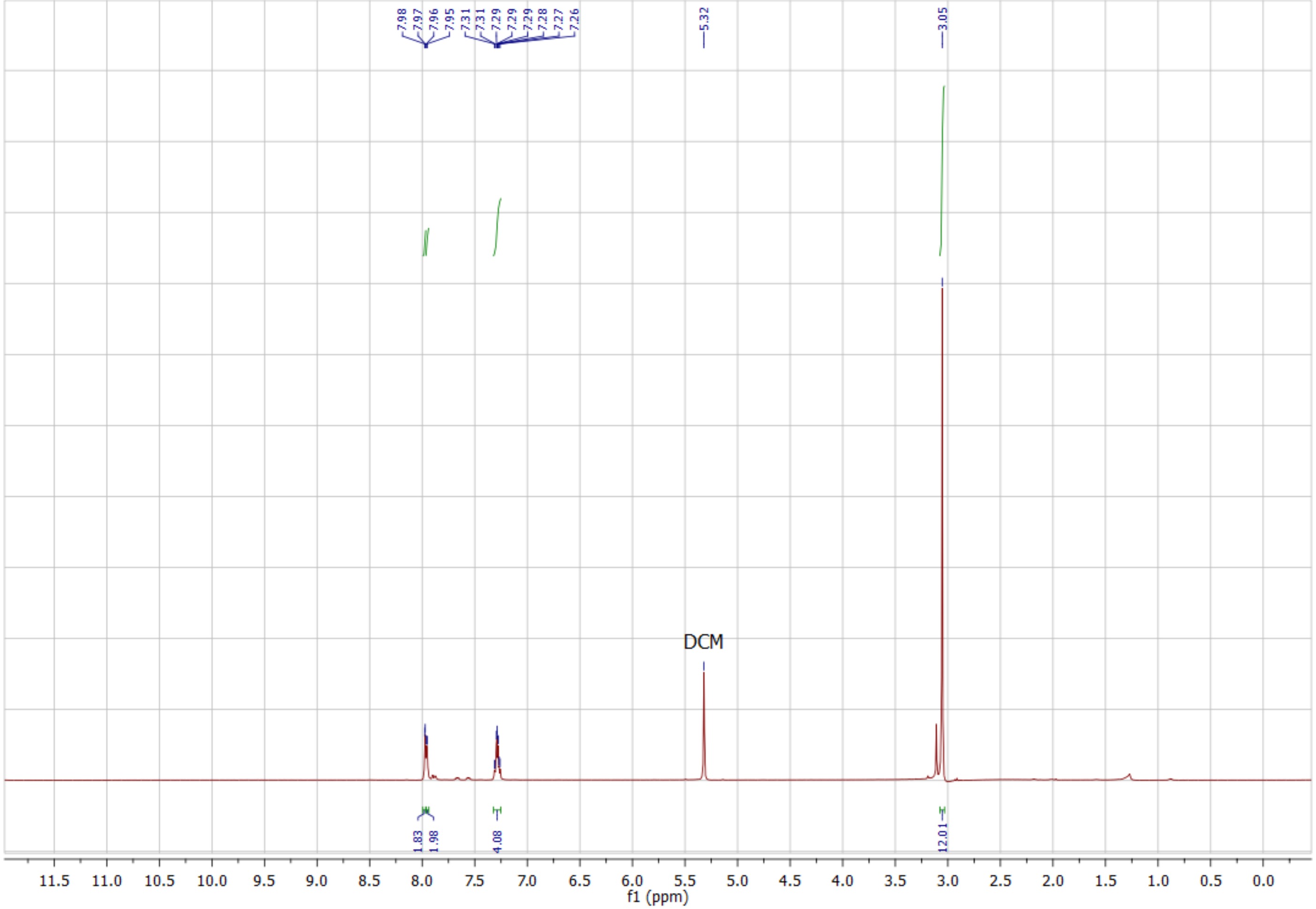

Figure S31: The ^{1}H NMR spectrum of complex **4b** in CD_2Cl_2.

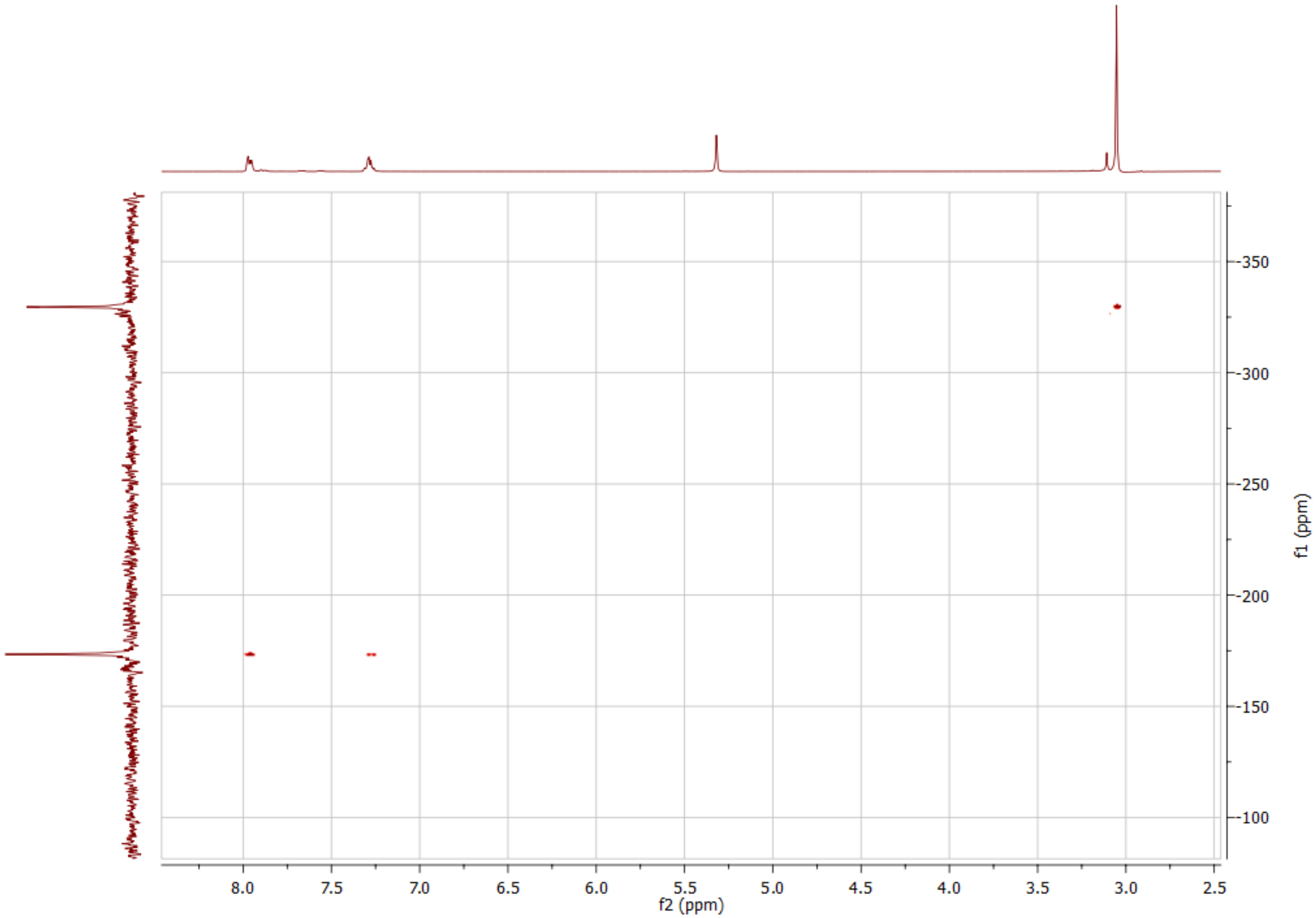

Figure S32: The ^{1}H-^{15}N HMBC spectrum of complex **4b** in CD_2Cl_2.

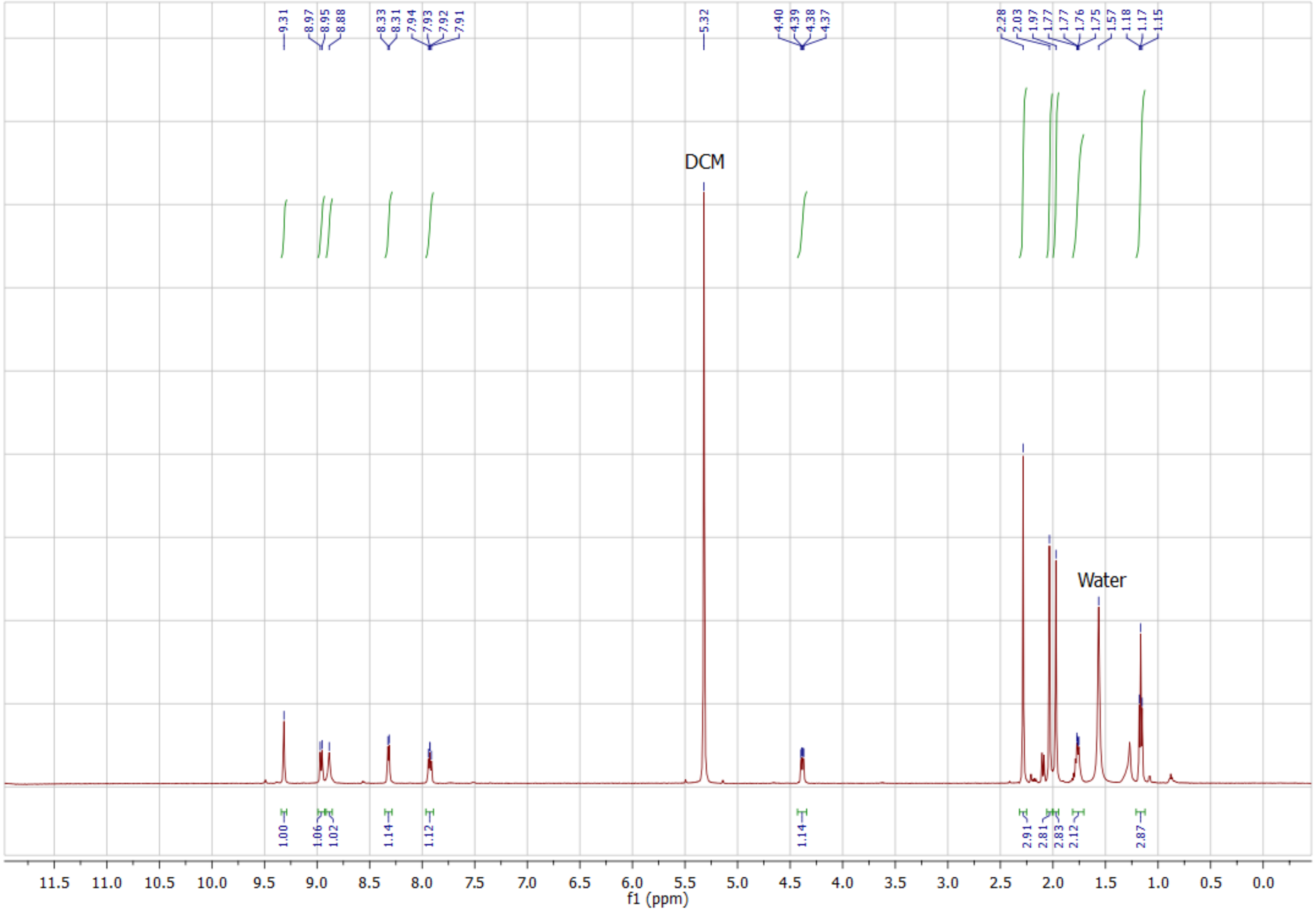

Figure S33: The ^{1}H NMR spectrum of complex **1d** in CD_2Cl_2.

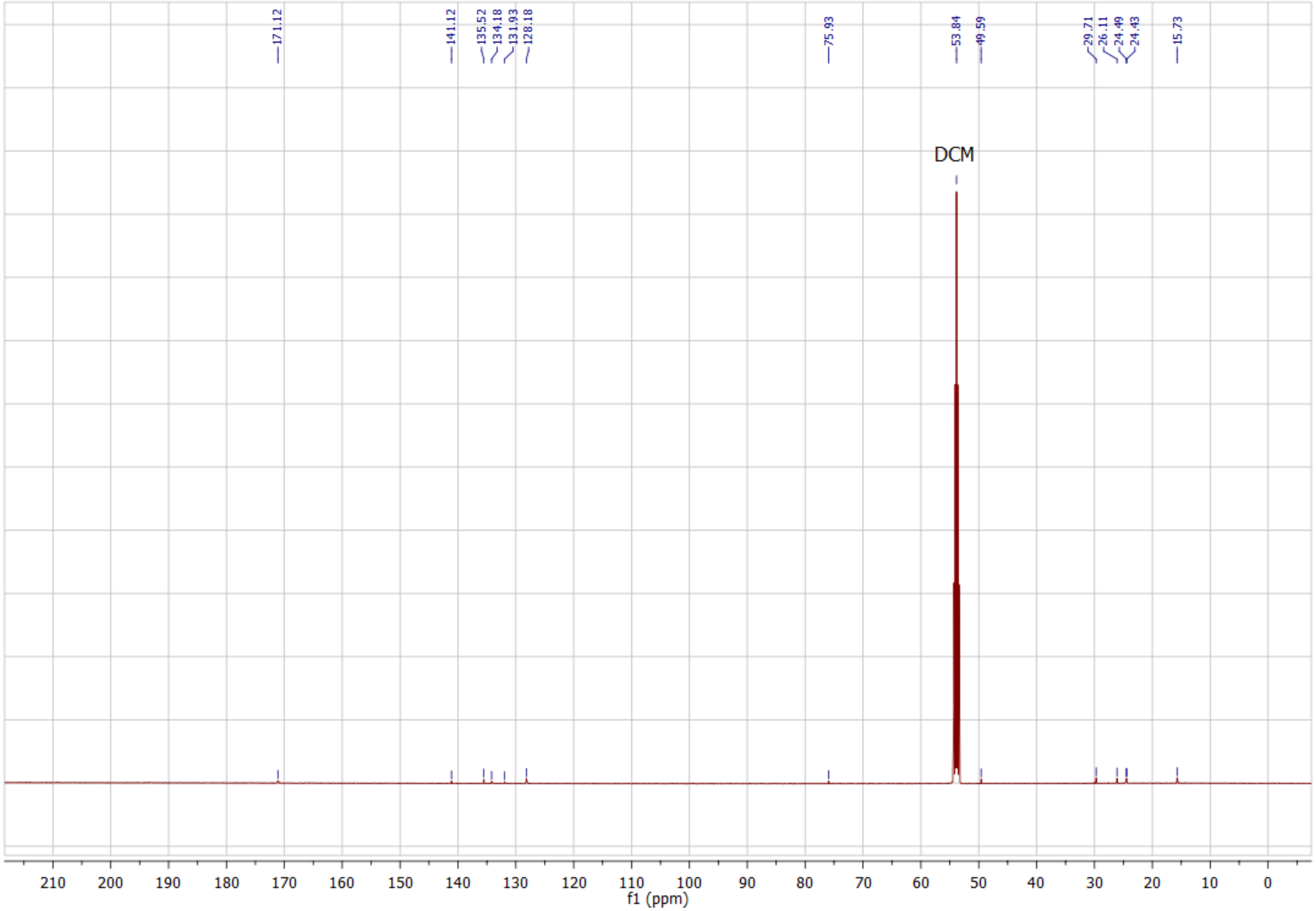

Figure S34: The ^{13}C NMR spectrum of complex **1d** in CD_2Cl_2.

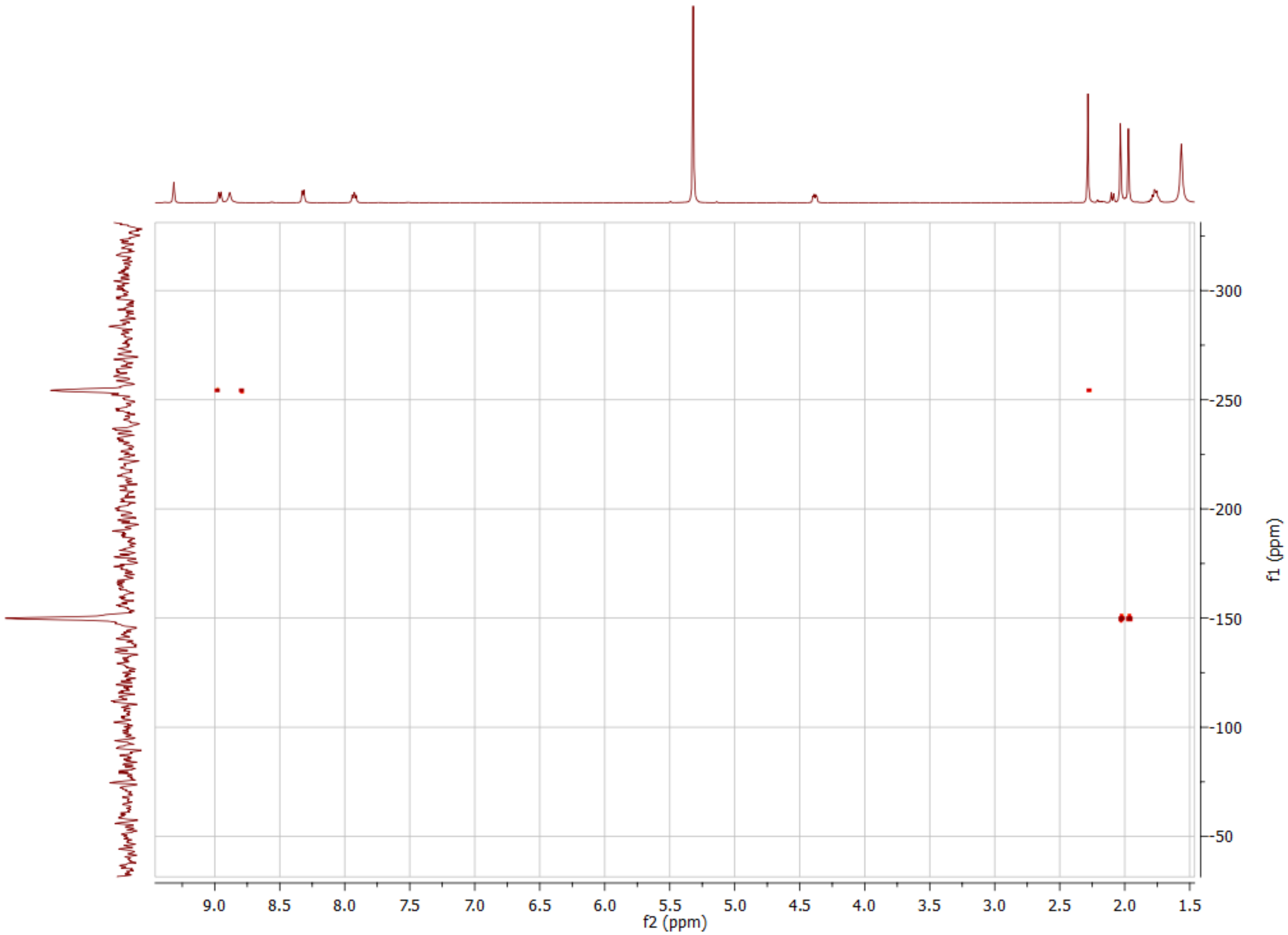

Figure S35: The ^{1}H-^{15}N HMBC spectrum of complex **1d** in CD_2Cl_2.

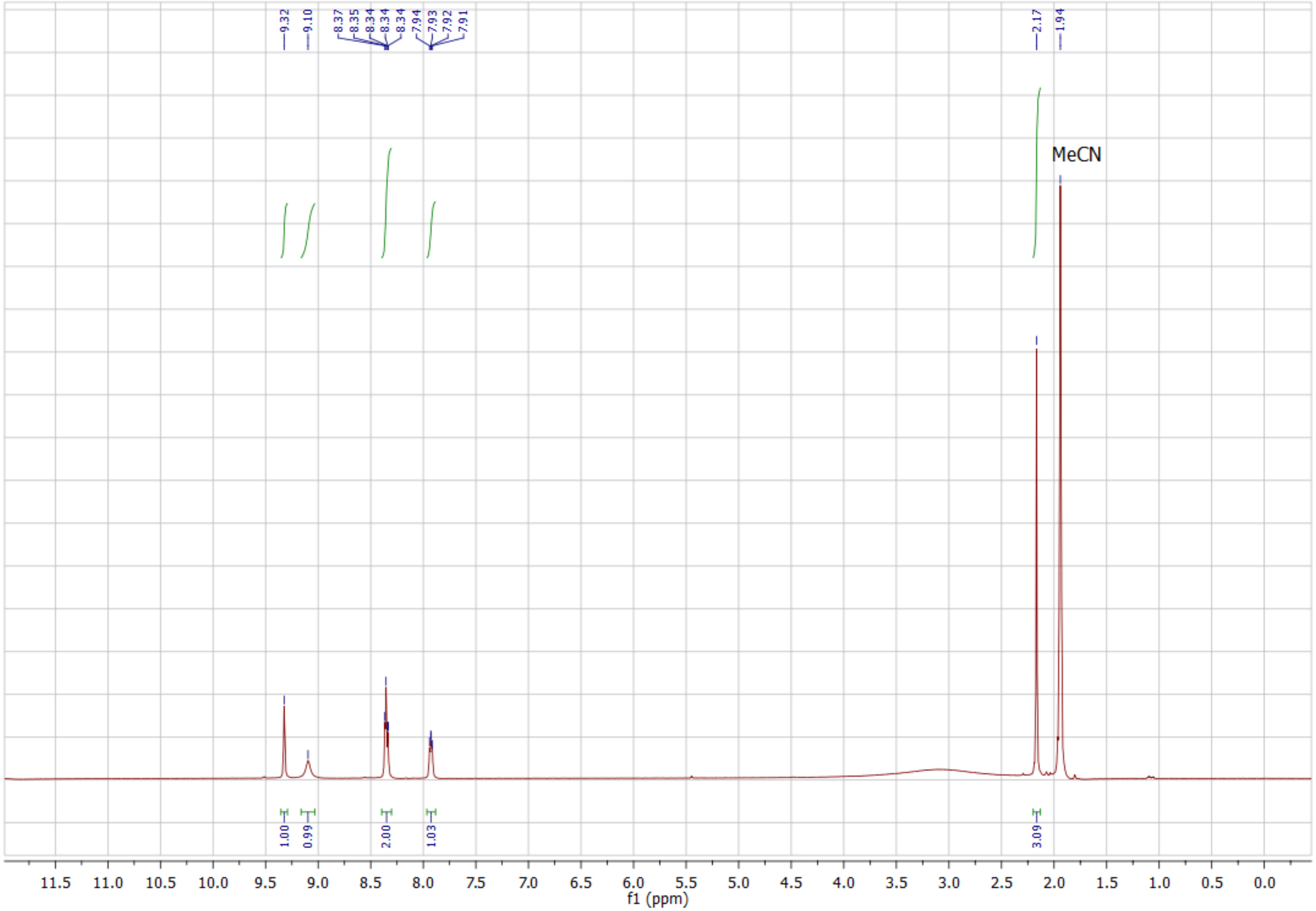

Figure S36: The ^{1}H NMR spectrum of complex **1e** in CD_3CN.

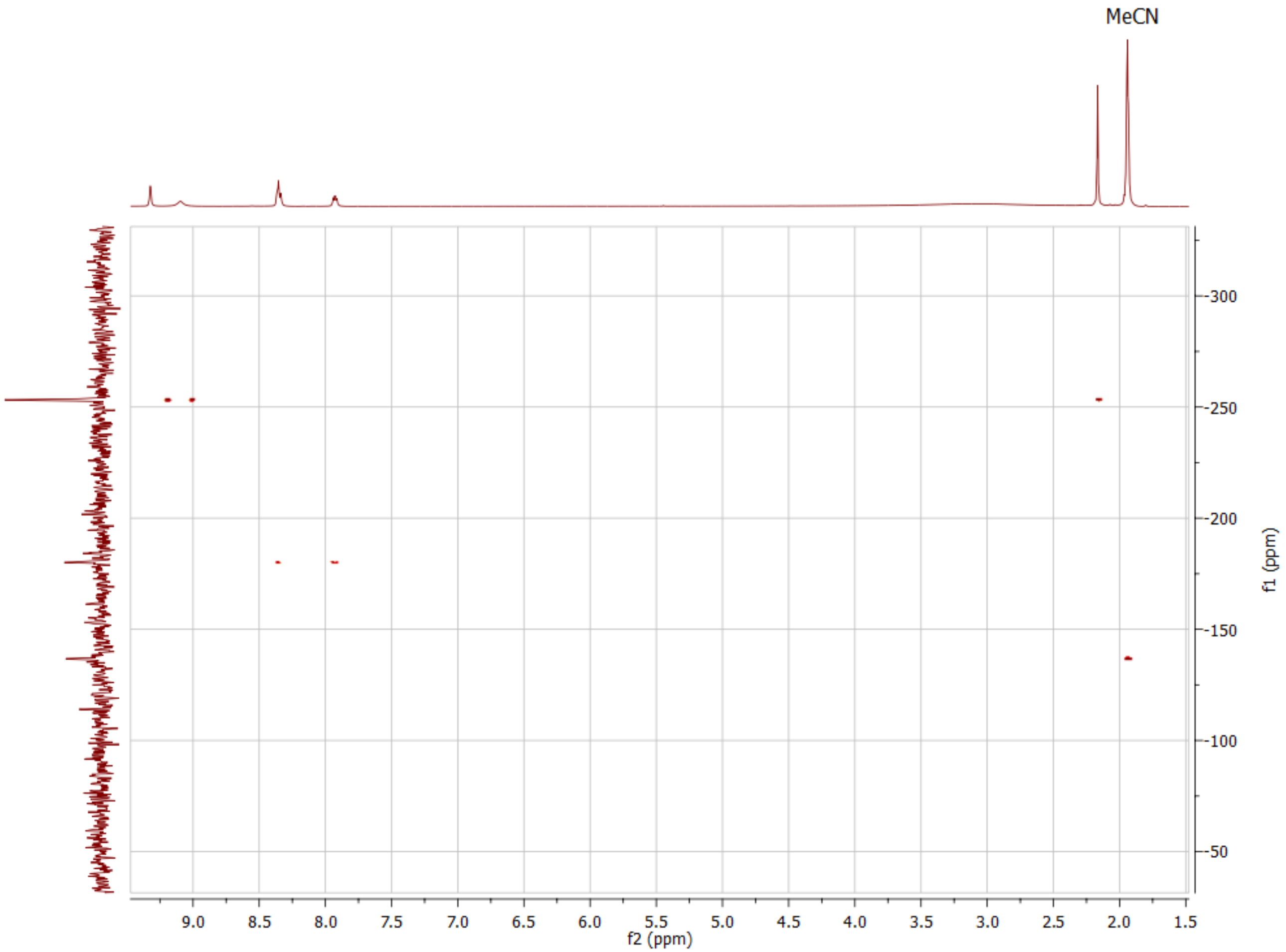

Figure S37: The ^{1}H-^{15}N HMBC spectrum of complex **1e** in CD_3CN.

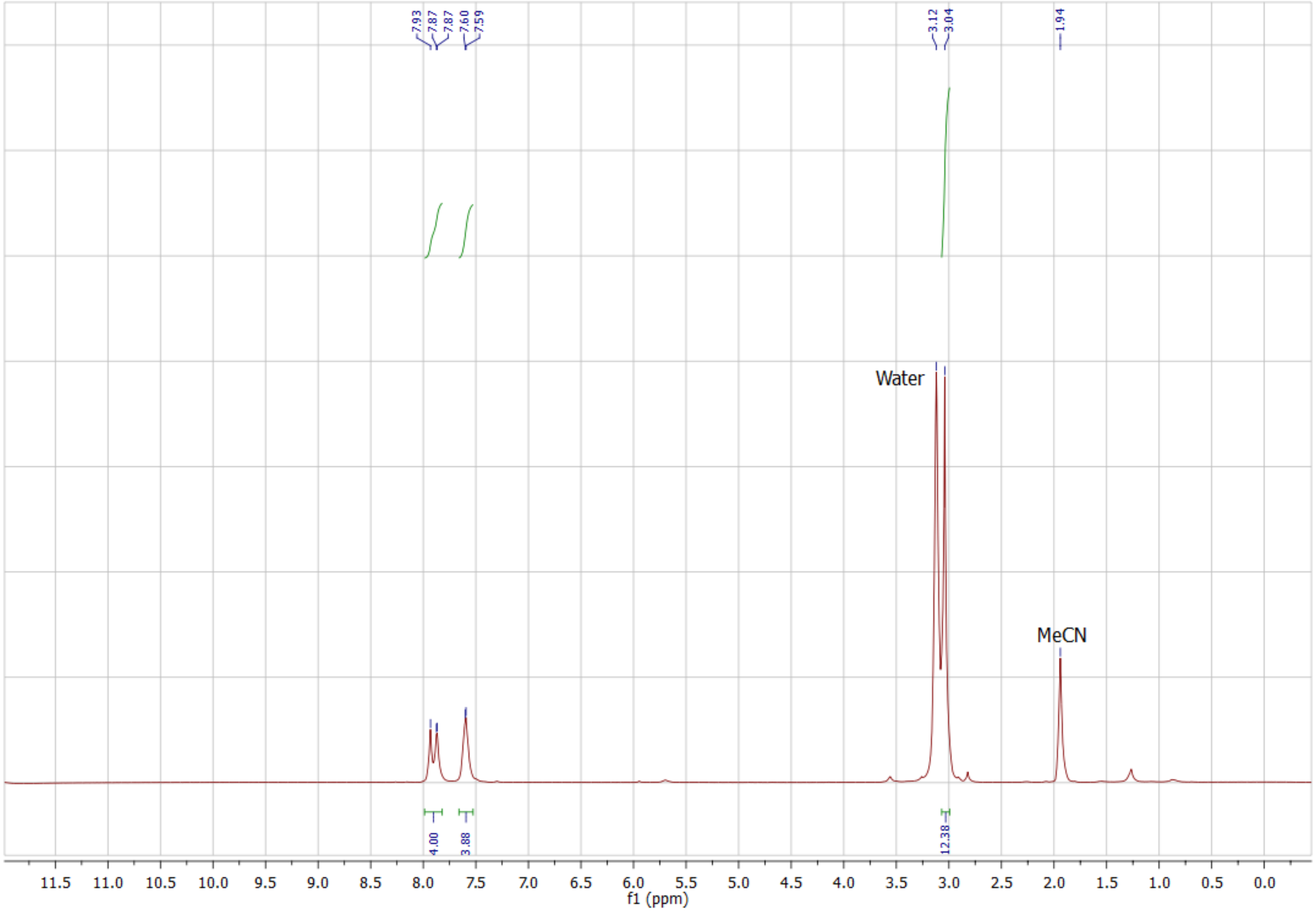

Figure S38: The ^{1}H NMR spectrum of complex **4f** in CD_3CN.

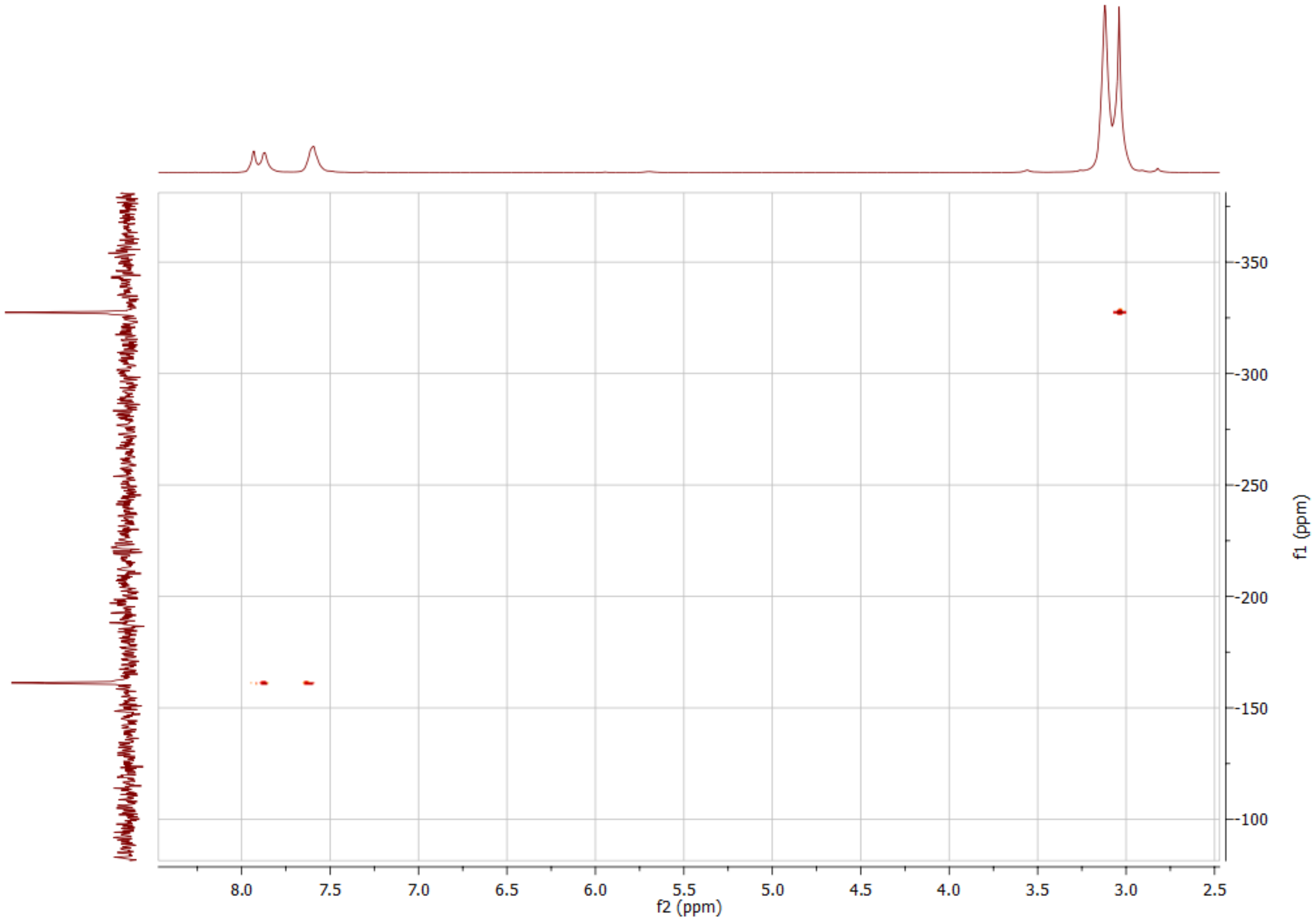

Figure S39: The ^{1}H-^{15}N HMBC spectrum of complex **4f** in CD_3CN.

References

(1) Ward, J. S. The Solid-State Hierarchy and Iodination Potential of [Bis(3-Acetaminopyridine)Iodine(i)]PF6. *CrystEngComm* **2022**, *24* (40), 7029–7033. https://doi.org/10.1039/D2CE01225A.

(2) Hooft, R. W. W.; Nonius. Collect. Nonius BV: Delft, The Netherlands 1998.

(3) Otwinowski, Z.; Minor, W. B. T.-M. in E. [20] Processing of X-Ray Diffraction Data Collected in Oscillation Mode. In *Macromolecular Crystallography Part A*; Academic Press, 1997; Vol. 276, pp 307–326. https://doi.org/https://doi.org/10.1016/S0076-6879(97)76066-X.

(4) Sheldrick, G. M. SHELXT – Integrated Space-Group and Crystal-Structure Determination. *Acta Crystallogr. Sect. A Found. Adv.* **2015**, *71* (1), 3–8. https://doi.org/10.1107/S2053273314026370.

(5) Dolomanov, O. V.; Bourhis, L. J.; Gildea, R. J.; Howard, J. A. K.; Puschmann, H. OLEX2 : A Complete Structure Solution, Refinement and Analysis Program. *J. Appl. Crystallogr.* **2009**, *42* (2), 339–341. https://doi.org/10.1107/S0021889808042726.

(6) Sheldrick, G. M. Crystal Structure Refinement with SHELXL. *Acta Crystallogr. Sect. C, Struct. Chem.* **2015**, *71* (Pt 1), 3–8. https://doi.org/10.1107/S2053229614024218.
